# X-Ray Microanalysis in the Variable Pressure (Environmental) Scanning Electron Microscope

**DOI:** 10.6028/jres.107.048

**Published:** 2002-12-01

**Authors:** Dale E. Newbury

**Affiliations:** National Institute of Standards and Technology, Gaithersburg, MD 20899-8371

**Keywords:** energy dispersive x-ray spectrometry, environmental scanning electron microscopy (ESEM), variable pressure scanning electron microscopy (VP-SEM), x-ray mapping, x-ray microanalysis, x-ray spectrometry

## Abstract

Electron-excited x-ray microanalysis performed in the variable pressure and environmental scanning electron microscopes is subject to additional artifacts beyond those encountered in the conventional scanning electron microscope. Gas scattering leads to direct contributions to the spectrum from the environmental gas, as well as remote generation of x rays by electrons scattered out of the focussed beam. The analyst can exert some degree of control over these artifacts, but depending on the exact situation, spurious elements can appear at the trace (< 0.01 mass fraction), minor (0.01 mass fraction to 0.1 mass fraction), or even major (> 0.1 mass fraction) levels. Dispersed particle samples give the least compromised results, while fine scale microstructures are the most severely compromised. Procedures to optimize the situation based upon specimen preparation as well as spectral processing are described.

## 1. Introduction

**Fig 1:**
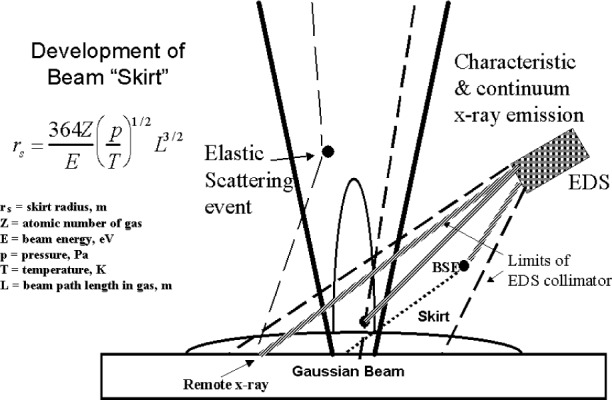
Schematic diagram illustrating formation of electron scattering “skirt” around the unscattered beam in a VPSEM-ESEM. Elastic scattering leads to transfer of electrons from the focused beam to the skirt. Inelastic scattering leads to inner shell ionization and subsequent emission of characteristic x rays from the gas, which will be collected by the EDS if emitted into the solid angle defined by the collimator.

Characterization of chemical microstructure is one of the most important applications of the conventional scanning electron microscope (SEM) equipped with an energy dispersive x-ray spectrometer (EDS) [1]. Interest in electron-excited x-ray microanalysis is potentially even greater in the variable pressure (VPSEM) and environmental scanning electron microscopes (ESEM) where dynamic chemical experiments can be conducted. The distinction between the VP-SEM and ESEM is made on the basis of achieving gas pressures that can maintain an equilibrium between water vapor and liquid water. With sample cooling to 2 °C, this equilibrium can be established at approximately 660 Pa. An arbitrary division between ESEM and VPSEM can be made at 100 Pa. X-ray microanalysis performed in the VPSEM-ESEM is subject to additional conditions and constraints that arise from the presence of the environmental gas and its influence on the primary electron beam. Figure 1 shows schematically the effects of elastic and inelastic scattering of the beam electrons by the gas atoms. The major consequence of inelastic scattering is the generation of characteristic and continuum (bremsstrahlung) x rays from the gas atoms that contribute to the measured Si-EDS spectrum. X-ray production is a relatively rare event, suffered by one in 10^6^ electrons or fewer. Most electrons do not suffer significant energy loss from inelastic interactions.

The consequences of elastic scattering are the reduction of beam current within the focused probe and redistribution of this current to form a wide “skirt” around the beam, significantly degrading the spatial resolution of x-ray microanalysis. These gas-scattering effects can greatly alter the results achieved with x-ray microanalysis in the VPSEM-ESEM compared to performing a similar x-ray measurement in a conventional SEM under high vacuum conditions, given that the specimen would be compatible with high vacuum. This paper will consider the special aspects of x-ray spectrometry and microanalysis performed in the VPSEM-ESEM, especially the impact of gas scattering on spectrum quality, methods of specimen preparation to minimize the effects of gas scattering, practical aspects of qualitative and quantitative x-ray microanalysis, and prospects for future improvements in this area.

## 2. X-Ray Spectrometry in the VPSEM-ESEM

Electron-excited x-ray spectrometry performed with wavelength dispersive spectrometry (WDS) and/or semiconductor energy dispersive spectrometry (Si-EDS) in the SEM is a mature technique that is widely employed across many of the sciences [1]. Specimen excitation with a focussed electron beam at a fixed position can achieve lateral spatial resolution down to approximately 1 µm or less, depending on the beam energy and the exact composition of the specimen at the beam location. WDS and Si-EDS have critical strengths and weaknesses (e.g., resolution, spectral coverage, limits of detection, speed of photon processing, etc.) that are mutually supportive, so that combined Si-EDS-WDS instruments represent the most sophisticated level of this instrumentation in conventional SEM applications [1]. Because of the constraints imposed by the more aggressive environment of the VPSEM-ESEM, virtually all x-ray spectrometry in these instruments has been performed with Si-EDS, usually equipped with a vacuum isolation window that is resistant to water vapor. WDS could, in principle, be incorporated, but the special optical focusing properties of WDS demand precise positioning of the electron-excited x-ray source, and the diffractors of the WDS would require special protection to avoid degradation from exposure to the environmental gas. In this paper, we will consider only Si-EDS for performing x-ray spectrometry in the VPSEM-ESEM.

After recording an x-ray spectrum at a fixed beam location, the x-ray microanalysis procedure consists of two distinct stages:

1. Qualitative analysis: The x-ray peaks are assigned to specific elemental constituents, and the broad categorization of major, minor, and trace is applied to each constituent so identified. These terms are defined (arbitrarily) as:
  Major: C > 0.1 mass fraction (> 10 weight percent)
  Minor: 0.01 ≤ C ≤ 0.1 mass fraction (1 to 10 weight percent)
  Trace: C < 0.01 mass fraction (< 1 weight percent)
2. Quantitative analysis: A numerical value is assigned to the concentration, along with a statistical measure of the precision as a measure of repeatability and of expected accuracy.

A separate procedure, x-ray mapping, involves measuring x-ray intensities while the beam is scanned in a regular array of locations to form an image that depicts the spatial distribution of elemental constituents.

In the following discussion, we must also be aware that Si-EDS conducted under conventional high vacuum conditions is itself subject to artifacts (e.g., escape peaks, coincidence or sum peaks, and remote excitation due to backscattered electrons and rescattering of BSEs in the specimen chamber) that must be understood and corrected to achieve optimum results. In the following discussion, an understanding of EDS artifacts in conventional high vacuum operation will be assumed. A large literature on SEM/EDS exists that describes all aspects of the measurement science of the technique, including spectral artifacts, peak identification, various mathematical peak modeling procedures for separating peak and background, accuracy of quantitative analysis, limits of detection, etc. (for comprehensive treatments, see Refs. [1,2,3]). This literature forms the basis for proceeding with Si-EDS in the VPSEM-ESEM.

Gas scattering of the primary beam is the single most important difference between performing x-ray spectrometry with the conventional low pressure (i.e., high vacuum) SEM and with the elevated pressure (low vacuum) VPSEM-ESEM instruments. X-ray spectrometry performed in the VPSEM-ESEM must inevitably be compromised because of gas scattering compared to the “ideal” situation in the conventional high vacuum SEM. The key problem to consider for practical microanalysis in the VPSEM-ESEM is determining the concentration level of the analyte in the specimen (major, minor, or trace) for which the results can be trusted.

### 2.1 Extraneous X-Ray Peak(s) Due to the Environmental Gas

X-ray spectrometry in the VPSEM-ESEM is subject to additional artifacts beyond those familiar in conventional SEM/EDS. These artifacts are directly related to the presence of the environmental gas. The inevitable gas scattering, both elastic and inelastic, of a fraction of the primary beam electrons has a significant and frequently severe impact on both qualitative and quantitative Si- EDS x-ray microanalysis in the VPSEM-ESEM. Considering first the case of inelastic scattering, both characteristic and continuum (bremsstrahlung) x rays are produced by the incident beam electrons during interactions with the environmental gas atoms. Moreover, the beam electrons that backscatter from the specimen can also undergo inelastic scattering events with the environmental gas atoms, further contributing to the measured x-ray spectrum. Although the density of atoms in the gas is very low compared to the atom density in the solid specimen, the volume of the gas which lies within the solid angle of collection of the Si-EDS, even when properly collimated, is quite large. The EDS accepts x rays from most of the gas path length of the beam from the final pressure limiting aperture to the specimen, a distance of several millimeters. The volume above the specimen into which the backscattered electrons are emitted (following a cosine distribution for a specimen surface placed normal to the beam) is also within the acceptance of the Si-EDS for the majority of BSEs, with only those lost which are emitted as a result of beam electrons scattered so far out into the skirt that they re-emerge as BSE outside the collimated acceptance area of the Si-EDS.

**Fig 2:**
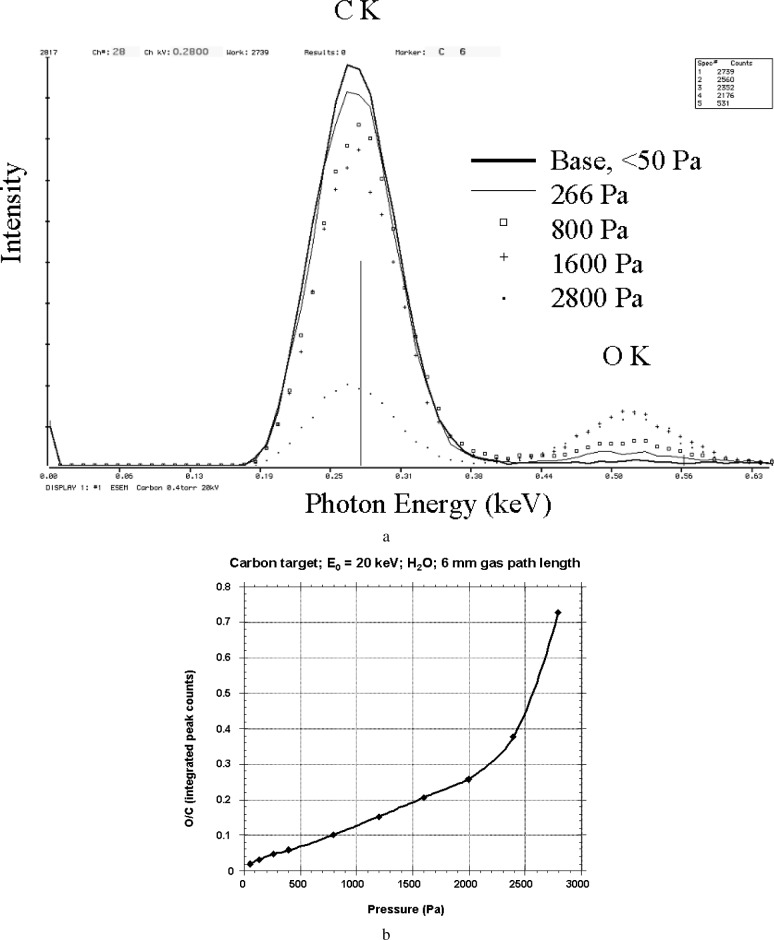
(a) Detection of x-ray emission from the environmental gas (H_2_O) at various pressures for a gas path length of 6 mm, an incident beam energy of 20 keV, normal incidence, and a 2.5 cm diameter carbon target: base: 53 Pa (0.4 torr); 266 Pa (2 torr); 1600 Pa (12 torr); 2800 Pa (21 torr); (b) plot of the x-ray intensity ratio O/C as a function of pressure.

Figure 2 (a) shows Si-EDS spectra obtained as a function of water vapor pressure from a pure carbon disk (2.5 cm in diameter) bombarded with 20 keV electrons with a beam gas path length of 6 mm. The artifact oxygen contribution is barely detectable at the base pressure (≈50 Pa), but develops into an easily detectable peak at 133 Pa (1 torr) and above. Figure 2 (b) shows a plot of the O/C peak intensity ratio as a function of the pressure. Depending on the pressure, the environmental gas can be detected in the x-ray spectrum as an apparent major, minor, or trace constituent relative to the legitimate peak from the target. At the highest pressure used (2800 Pa = 21 torr), the oxygen peak intensity reached more than 70 % of the C K peak intensity from the carbon target. Close examination of Figure 2 (a) reveals that initially the oxygen intensity increases with increasing water vapor pressure with a gradual lowering of the carbon peak relative to the measurement at base pressure. At the highest pressure, the carbon peak is substantially reduced in intensity compared to the base level. This reduction in carbon x-ray intensity occurs because of elastic scattering into the skirt at distances beyond the acceptance of the EDS collimator (see next section) rather than from energy loss. Below approximately 10 Pa (0.1 torr), the contribution of the environmental gas to the x-ray spectrum becomes negligible. When He-H_2_ gas mixtures are used instead of H_2_O or air, extraneous x rays from the gas can be eliminated because of the lack of measurable x-ray emission from these atoms. There is still a contribution to the composite spectrum from continuum x rays produced from this gas mixture. For equivalent gas densities, the intensity of this extraneous continuum radiation contribution will be lower for He-H_2_ because of the proportional dependence of the continuum on atomic number.

Does the environmental gas act to significantly absorb the x rays emitted from the specimen? Table 1 gives the results of calculations of absorption for various photon energies as a function of pressure (2500 Pa, 100 Pa, and 10 Pa) for oxygen as the environmental gas with a specimen-to-EDS path length of 4 cm. For the VPSEM pressure range (10 Pa and 100 Pa) x-ray absorption by the gas phase is not a significant effect, being only about 6 % for F K x rays, which are strongly absorbed by oxygen. For the upper end of the ESEM pressure range (2500 Pa), absorption is a significant effect, attenuating F K by 81 %, NaK by 43 %, AlK by 20 %, and S K by 6 % for a 4 cm specimen to EDS pathlength through the environmental gas.

### 2.2 Primary Beam Gas Scattering: Remote Excitation of X Rays

The electrons scattered out of the beam by elastic interactions with the atoms of the gas form a broad, non-focused “skirt” around the unscattered, focused portion of the primary beam. Danilatos (1988) has described the development of the skirt with the following equation [4]:

$$r_{s}=\left(\frac{364Z}{E}\right){\left(\frac{p}{T}\right)}^{1/2}L^{3/2}$$ (1)

where

*r*_s_ = skirt radius, m
*Z* = atomic number of the gas
*E* = beam energy, eV
*P* = pressure, Pa
*T* = temperature, K
*L* = beam path length in gas, m

**Fig. 3:**
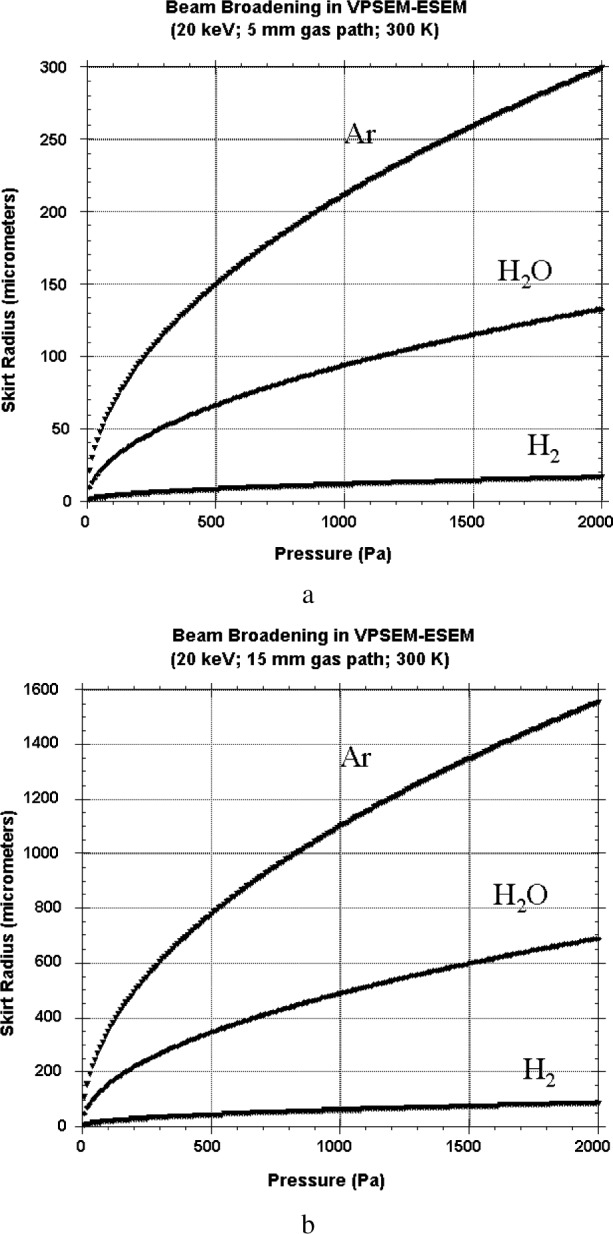
Development of scattering skirt as calculated with Eq. (1) (Fig. 1) at a beam energy of 20 keV for various gases and a gas path length of (a) 5 mm; (b) 15 mm.

Figure 3 shows examples of the skirt radius calculated with Eq. (1) for various gases (hydrogen, water vapor, and argon) for a beam energy of 20 keV and gas path lengths of 5 mm [Fig. 3 (a)] and 15 mm [Fig. 3 (b)]. The scale (in linear dimensions) of the skirt relative to the beam can cover many orders of magnitude. Depending upon the beam energy, environmental gas species and pressure, and the gas path length, the skirt can have a diameter of millimeter or more [e.g., in Fig. 3 (b), Ar above a pressure of 800 Pa gives a skirt radius above 1 mm], while the focused beam diameter may be 10 nm or less, giving a skirt/beam ratio of 10^5^. What is the influence of the skirt on electron imaging and x-ray spectrometry performed in the VPSEM and ESEM?

While a large fraction, 50 % or more, of the beam current can be transferred from the focused beam to the skirt due to gas scattering, the electron current density (A/cm^2^) at any point in the skirt is much lower than that in the focussed beam. When a conventional electron image is formed by scanning such a beam/skirt combination over an array of pixels, it is the high current density of the focused beam that produces sharp responses across fine details of the specimen topography and creates a high resolution image. The skirt, which can even contain the majority of the beam electrons, is so spread out and locally diffuse that during a scan, particularly at high magnification, the skirt barely moves relative to the fine features that form the interesting image details. The gas-scattered skirt component is so widely spread that the electron imaging signals it produces are completely decoupled from the local imaging environment experienced by the focused beam. Thus, the skirt merely adds a non-specific, steady state (DC) level to the measured signal which does not vary significantly with the scan position of the primary focused beam. Local topography on the scale of the beam influences the beam related signal only. The principal effect of the skirt signal on image quality is to increase the statistical noise upon which the useful scan varying signal information rides, lowering the signal-to-noise (*S*/*N*) ratio. A deterioration in the *S*/*N* ratio raises the minimum level of feature contrast that can be rendered visible in the SEM image. This negative impact on signal quality can usually be overcome by increasing the pixel dwell time so that adequate images can be obtained even from low contrast features. Useful electron imaging with gas scattering losses as high as 90 % of the total current has been reported, provided enough accumulation time is used [4]. Thus, although gas scattering to form the beam skirt is deleterious to electron imaging, the interaction of the skirt electrons with the sample can be largely ignored and high quality, high resolution electron images can be regularly obtained with the VPSEM-ESEM.

**Fig. 4:**
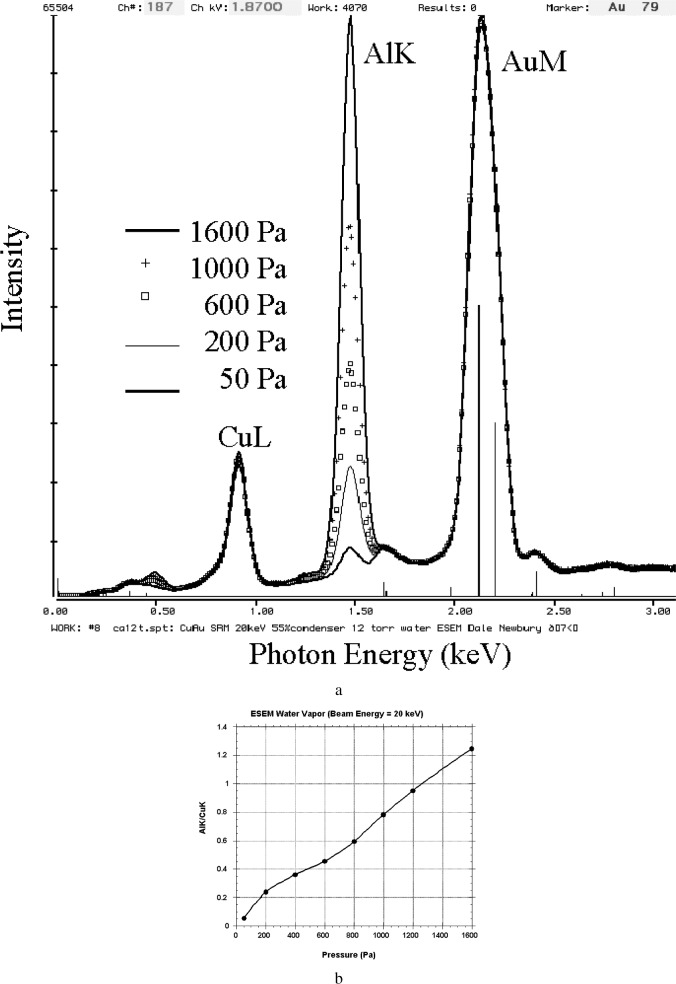
Remote scattering of beam electrons. Target: 500 µm diameter wire of 40Cu-60Au (selected from NIST Standard Reference Material (SRM) 482 Copper-Gold Alloys) embedded in a large (2.5 cm diameter) aluminum disk; beam energy 20 keV; normal incidence. Pressure: 50 Pa (0.4 torr); 200 Pa (1.5 torr); 600 (4.5 torr); 1000 Pa (7.5 torr); 1600 Pa (12 torr); (b) Plot of x-ray intensity ratio Al-K/Cu-K vs pressure.

When we consider the impact of the beam skirt on x-ray spectrometry in the VPSEM-ESEM, we encounter a much different circumstance. The remotely scattered skirt electrons interact with the specimen atoms that they encounter, producing the characteristic and continuum x rays appropriate to each location. If these x rays produced by the skirt electrons are within the solid angle of acceptance of the x-ray spectrometer, they are indistinguishable from the characteristic and continuum x rays produced by the focussed probe within its interaction volume. The x-ray spectrum thus measured is actually a composite spectrum with contributions from the beam and the skirt, but the analyst has no immediate way to distinguish which x rays are from the focussed beam and which are from the skirt. This effect is illustrated in Fig. 4, which shows Si-EDS spectra recorded on a 500 µm diameter wire of 40Cu-60Au (nominal mass fraction, selected from NIST Standard Reference Material (SRM) 482 Copper-Gold Alloys) embedded in a large (2.5 cm diameter) aluminum disk [5]. For a fixed beam gas path length of 2 mm, the skirt contribution on the aluminum holder increases as the pressure of the water vapor increases, as shown in the sequence of spectra superimposed Fig. 4 (a): 53 Pa (0.4 torr); 4 (b) 200 Pa (1.5 torr); and 4 (c) 1600 Pa (12 torr). Plotting the contribution of the AlK peak to the spectrum as the ratio AlK/CuK produces the plot as a function of pressure shown in Fig. 4 (b). A nearly linear response is observed for AlK/CuK vs pressure. This near-linear behavior is a consequence of the circularly symmetric geometry of the wire-disc composite target. From Eq. (1), the skirt radius *r* ≈ *p*^1/2^. The area of the skirt, which is proportional to *r*^2^, is therefore proportional to *p*. Linear behavior vs pressure in the skirt component will therefore be observed providing the gas scattered electrons strike common material, which is the situation for the circular symmetry target. The linear behavior vs pressure is eventually lost at high pressures because gas scattering takes so much current out of the beam striking the alloy wire that the peak intensities of the alloy components begin to decrease significantly.

The sequence of spectra shown in Fig. 4 reveals that, depending on the degree of gas scattering, the pathological peak due to the contribution to the composite spectrum of the remote scattering onto the surrounding matrix can have the appearance of a trace, minor, or major constituent, Fig. 4 (a). Gas scattering can profoundly influence the interpretation of a spectrum and thus affect both stages of x-ray microanalysis: (1) qualitative analysis wherein the peaks from gas scattering are assigned to elemental constituents not actually present in the specimen sampled by the unscattered beam, and (2) quantitative analysis, in which the elements contributed by gas scattering will alter the matrix correction calculation by introducing unnecessary corrections for absorption, etc. Observing the composite spectrum obtained from specimens with complex microstructures, such as a fine scale discontinuous phase in a matrix phase, some early researchers quickly became dismayed with the prospects for useful x-ray microanalysis in VPSEM-ESEM and dismissed the technique prematurely [6; E. Lifshin, State University of New York at Albany; private communication]. Restricted as they were to using long gas paths in this early instrumentation, it is not surprising that these early workers reached this conclusion.

**Fig. 5:**
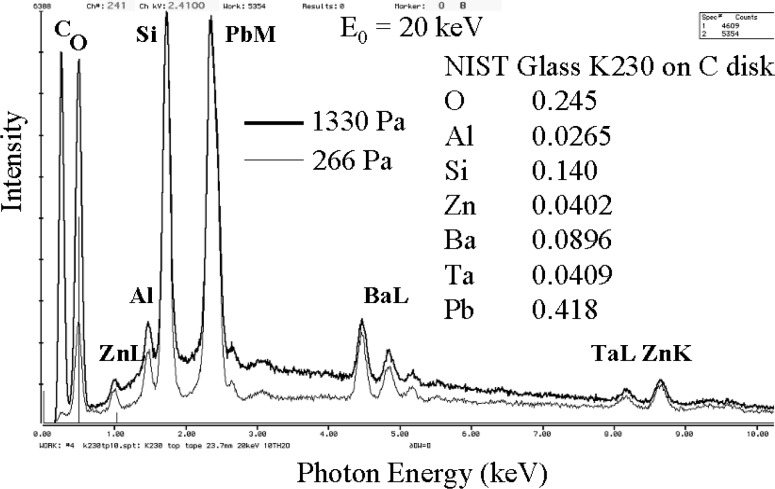
Spectra of a particle of NIST Glass K230 (approximately 50 µm in dimension) on a carbon disk at pressures of 266 Pa (2 torr) and 1330 Pa (10 torr) showing increase in the skirt contribution from the carbon as well as direct excitation of the environmental gas (water vapor) by beam and backscattered electrons.

Both elastic and inelastic gas scattering effects can be recognized in the same spectrum. Figure 5 shows a spectrum of an NIST glass (K-230) measured as a small fragment (approximately 50 µm in dimensions) placed on a large (2.5 cm diameter) carbon planchet at two different pressures, 266 Pa and 1330 Pa [5]. Inelastic scattering can be recognized as the increase in the relative intensity of the oxygen K-peak due to the water vapor used as the environmental gas in spectrum measured at the higher pressure. Elastic scattering manifests itself in the relative increase in the carbon K-peak in the spectrum measured at the higher pressure. A second, less obvious effect of elastic scattering is the increase in the continuum background and the lower peak-to-background ratio of the various spectral peaks (e.g., ZnL, AlK, SiK, PbM, etc.) caused when gas scattering removes electrons that interacted with the glass fragment at the lower pressure but which strike the carbon planchet at higher pressure, generating carbon K-peak x rays and continuum at all other photon energies.

### 2.3 Charging Effects

One of the principal strengths of the VPSEM-ESEM is the possibility of examining of insulating materials without the necessity of modifying the surface with a conductive coating, as must be done for the conventional high vacuum SEM. In the VPSEM, the inelastic scattering of beam and backscattered electrons with gas atoms creates free electrons and positive ions. These charged species are automatically attracted to charged areas on the specimen surface, thus acting to discharge them. In the ESEM, the gas ionization process is further augmented by secondary electrons, emitted from the specimen surface by inelastic scattering of beam and backscattered electrons, that are subsequently accelerated by the applied potential of the gaseous secondary electron detector. A cascade of ionization and secondary electron multiplication creates a much higher density of charge carriers in the ESEM. Stable images of bare insulators, even with deep holes, can be observed with this form of charge compensation.

**Fig. 6:**
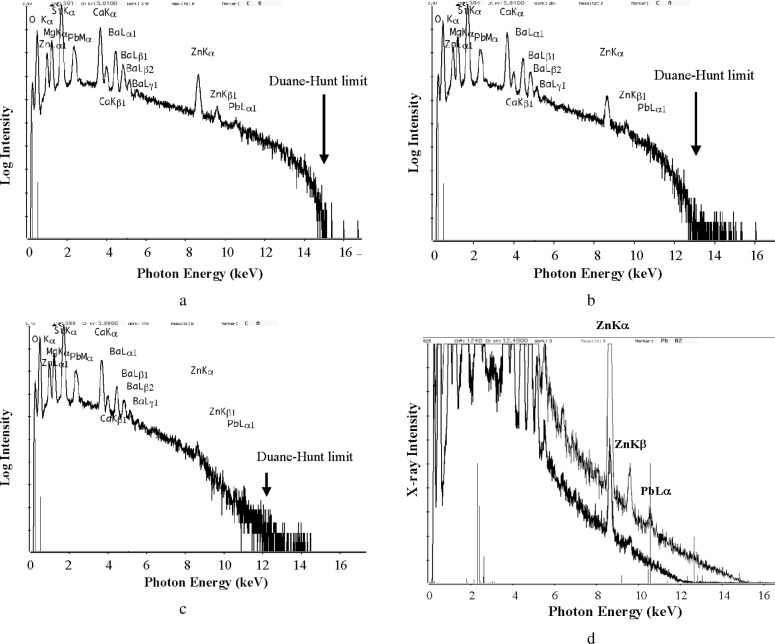
Charging effects observed with NIST glass 1070 (Mg = 0.075 mass fraction; Si = 0.0187 mass fraction; Ca = 0.0893 mass fraction; Zn = 0.01 mass fraction; Ba = 0.0112 mass fraction; Pb = 0.0928 mass fraction; O = 0.0343 mass fraction) under various environmental gas conditions. (a) 266 Pa (2 torr) showing the correct Duane-Hunt limit at 15 keV; (b) 67 Pa (0.5 torr) showing reduced intensity near the Duane Hunt limit and a limit of 13 keV; (c) 53 Pa (0.4 torr) showing a Duane Hunt limit of 12 keV; (d) Comparison of spectra recorded at 266 Pa (2 torr) and 53 Pa (0.4 torr) with a linear intensity scale, showing loss of PbLα due to charging, and severe reduction in intensity for ZnKα and ZnKβ.

A full understanding of charge control in the VPSEM-ESEM imaging process continues to evolve. However, even if a stable image of an insulator can be obtained, the analyst must still be wary that charging effects can influence the x-ray spectrum. The Duane-Hunt limit, the continuum energy that corresponds to the energy of the incident beam as it reaches the surface of the specimen, is a useful diagnostic to detect charging conditions [1]. Figure 6 shows a sequence of EDS spectra measured on an NIST glass, K1070 (Mg = 0.0750 mass fraction; Si = 0.0187 mass fraction; Ca = 0.0893 mass fraction; Zn = 0.0100 mass fraction; Ba = 0.0112 mass fraction; Pb = 0.0928 mass fraction; O = 0.0343 mass fraction) as a function of pressure from 266 Pa (2 torr) down to 53 Pa (0.4 torr) [6]. The spectra are displayed with a logarithmic intensity axis which makes determination of the Duane-Hunt limit straightforward by extrapolating the high energy continuum to its intersection with the energy axis at 0 intensity. (Note that there will always be some counts above the true Duane Hunt limit due to pulse pile-up of lower energy photons.) At 266 pA (2 torr), the spectrum in Fig. 6 (a) shows a Duane Hunt limit of 15 keV, which is equivalent to the beam energy selected, indicating that the gas/ion/electron environment of the VPSEM-ESEM is successfully preventing the accumulation of charge on the specimen surface. When charge builds up on the surface, it acts to accelerate the electron beam. If this charge is negative, as is usually the case for an electron beam incident on an insulating surface, then the negative charge will act to slow the incoming electron velocity so that the landing energy is less than the electron gun energy. This effect is seen in Fig. 6 (b), which shows the spectrum obtained at 67 Pa (0.5 torr) where the Duane-Hunt limit has dropped to approximately 13 keV, indicating surface charging to approximately 2000 V. Lowering the pressure to 53 Pa (0.4 torr) in Fig. 6 (c) leads to a further decrease in the Duane Hunt limit to approximately 12 keV. Close examination of Figs. 6 (b) and 6 (c) shows photons recorded between the “real” beam energy and the charge-limited Duane Hunt condition; these photons are too abundant to be solely due to pulse coincidence. These photons are indicative of the dynamic nature of charging, where there may be a momentary discharge of the accumulated electrons, followed by a rapid build-up. During the time of the discharge when the beam electrons are not decelerated, a few photons may be created near the true Duane-Hunt limit, but most of the spectrum is dominated by charging. For the spectrum of K1070, the effect of this charging is to lose the high energy characteristic x-ray peaks for Zn K and Pb L, as shown in Fig. 6 (d) with a linear intensity axis for spectra recorded at pressures of 266 Pa (2 torr) and 53 Pa (0.4 torr) with a linear intensity axis. The PbLα peak is lost entirely due to charging, and ZnKα and ZnKβ are also reduced in intensity.

Charging effects such as those illustrated in Fig. 6 can have a catastrophic impact on both qualitative and quantitative x-ray microanalysis by severely modifying the relative peak heights and in some cases completely suppressing high energy peaks. The analyst performing x-ray microanalysis in the VPSEM-ESEM needs to be extremely careful when measuring x-ray spectra from uncoated, insulating specimens. Time dependent, dynamic charging situations are likely to occur. To detect dynamic charging during spectrum accumulation, it is useful to have within the EDS control software the capability to define a series of “ratemeters” covering specified energy ranges that are placed throughout the x-ray spectrum (covering peaks and/or continuum windows), especially in the high photon energy range. If there is no charging, the counting rates should be constant, except for normal statistical fluctuations, in all windows throughout the spectral accumulation. When charging occurs, it should affect the higher photon energy windows near the Duane-Hunt limit first.

## 3. Strategies for Dealing With Gas Scattering

Several strategies have been developed for dealing with gas scattering and the inevitable excitation of portions of the specimen remote from that being interrogated by the direct beam [7]. These strategies depend on the type of specimen to be examined and the level of compositional information sought. However, it must be recognized from the outset that x-ray spectrometry performed in the VPSEM-ESEM can never be as good as that performed in the high vacuum SEM. Nevertheless, the value of x-ray microanalysis in VPSEM-ESEM studies can be so great that it is worth the analyst’s efforts to implement additional specimen preparation protocols and analysis procedures to achieve the best possible results within the limitations imposed by gas scattering.

### 3.1 Selection of Instrumental Parameters to Minimize Gas Scattering

Equation (1) describes the impact of various parameters on elastic scattering by the gas atoms. Thus, the size of the skirt and the extent of remote scattering can be reduced by:

1. increasing the beam energy, which decreases the elastic scattering probability;
2. reducing the average atomic number of the environmental gas, e.g., using oxygen instead of argon, helium-hydrogen instead of oxygen);
3. reducing the pressure of the environmental gas to the minimum consistent with stable operation or with the requirements of the experiment;
4. increasing the temperature;
5. decreasing the gas path length.

From the exponents on the various terms in equation (1), we can see that modifying the gas path length will have one of the strongest influences on the skirt and remote scattering.

Of course, the experimentalist may not have the latitude to change specific parameters over the full possible range. For example, there may only be a narrow range of chamber pressure over which stable charge-free imaging can be achieved, or the particular chemical reaction under study may require a specific gas species, pressure and temperature to obtain the desired kinetics. Thus, to conduct a particular experiment in the VPSEM-ESEM, the gas path length and the beam energy may be the only variables with which to work.

Recognition of the importance of the gas path length in controlling the skirt diameter has led to important advances in the design of the VPSEM-ESEM, especially for operation in the highest pressure regime [4,7]. For the ESEM-class of instruments, which are capable of operating in the pressure range of 100 Pa to 2500 Pa (≈1 torr to 20 torr) or higher, gas scattering is especially significant, and reducing the gas path length to the practical minimum is necessary to preserve as much spatial resolution as possible. To compensate for the longer working distance needed to accommodate a side-mounted EDS detector while minimizing the gas path length, the low pressure beam path has been extended into the specimen chamber through the use of a conical pressure limiting aperture with a large altitude-to-diameter ratio. In addition, by bending the side-loading EDS snout through a large angle from the horizontal, typically 40° to 50°, then a large positive x-ray take-off angle, 25° to 50°, can be achieved even when the specimen is oriented so that the beam is placed normal to the surface.

For x-ray microanalysis, the choice of beam energy *E* is frequently constrained by the elemental species to be measured. The generation of characteristic x rays depends strongly on the overvoltage, which is the ratio of the incident beam energy (*E*_0_) to the critical excitation or edge energy (*E*_c_), *U* = *E*_0_/*E*_c_. The characteristic intensity *I* ≈ (*U* − 1)*^n^*, where 1.3 ≤ *n* ≤ 1.6 [1]. Generally good analysis practice requires an overvoltage of at least *U* = 2 to produce adequate characteristic peak intensity above the continuum (bremsstrahlung) background. The photon energy range from 0.1 keV to 12 keV provides a K, L, and/or M shell x-ray for all elements in the Periodic Table with *Z* ≤ 4 (Be). For conventional microanalysis performed in high vacuum systems, a general rule is to select an incident beam energy that is a factor of 2 greater than the most energetic elemental excitation edge to be measured. The choice of a high primary beam energy, 20 keV or more, provides efficient excitation of the upper part of the photon energy range, and is a good choice for the VPSEM-ESEM since a high beam energy also serves to minimize gas scattering. However, a negative consequence of a high incident beam energy is the relative reduction in intensity of low photon energy x rays (≤ 3 keV) due to increased absorption within the specimen. Absorption occurs because the x rays are generated deeper into the specimen as a result of the greater range of the beam electrons, which increases approximately as *E*_0_^1.66^. The strategy of lowering the beam energy to reduce the electron range and to lower the absorption losses for low energy photons that is available in conventional high vacuum operation may be severely restricted in VPSEM-ESEM because of the rapid increase in gas scattering as the beam energy decreases.

### 3.2 Pressure Variation Method

Doehne and Bilde-Sorenson and Appel have suggested a method of estimating the skirt contribution to the spectrum through measurements at different pressures to predict the spectrum that would be obtained with no gas scattering [8,9,10]. As described in detail by Doehne, this method requires recording two spectra with all other conditions identical except for the pressure [8]. If “A” is the spectrum recorded at higher pressure and “B” the lower, then the “zero scattering” spectrum “C” is estimated by:

C=B−[(A−B)⋅d] (2)

where “d” is an empirical scaling factor. Implicit in this method is the assumption that changes to the skirt contribution with pressure are primarily due to the skirt intensity rather than the extent of the skirt, that is, the compositional environment excited by the skirt electrons does not change significantly as the extent of the beam skirt increases with increasing gas scattering due to increased pressure [11]. If a peak is entirely contributed by the skirt (i.e., the “true” spectrum only arises from the focussed beam), then there will be a distinct change is intensity of the skirt peak(s) as the pressure is changed. Doehne (1997) has demonstrated the capability of eliminating minor intensity skirt peaks by this method [8]. Note that because difference methods are used, it is very important to obtain high count spectra, so that the variance in the spectra does not dominate low intensity peaks of interest in the processed spectrum.

**Fig. 7:**
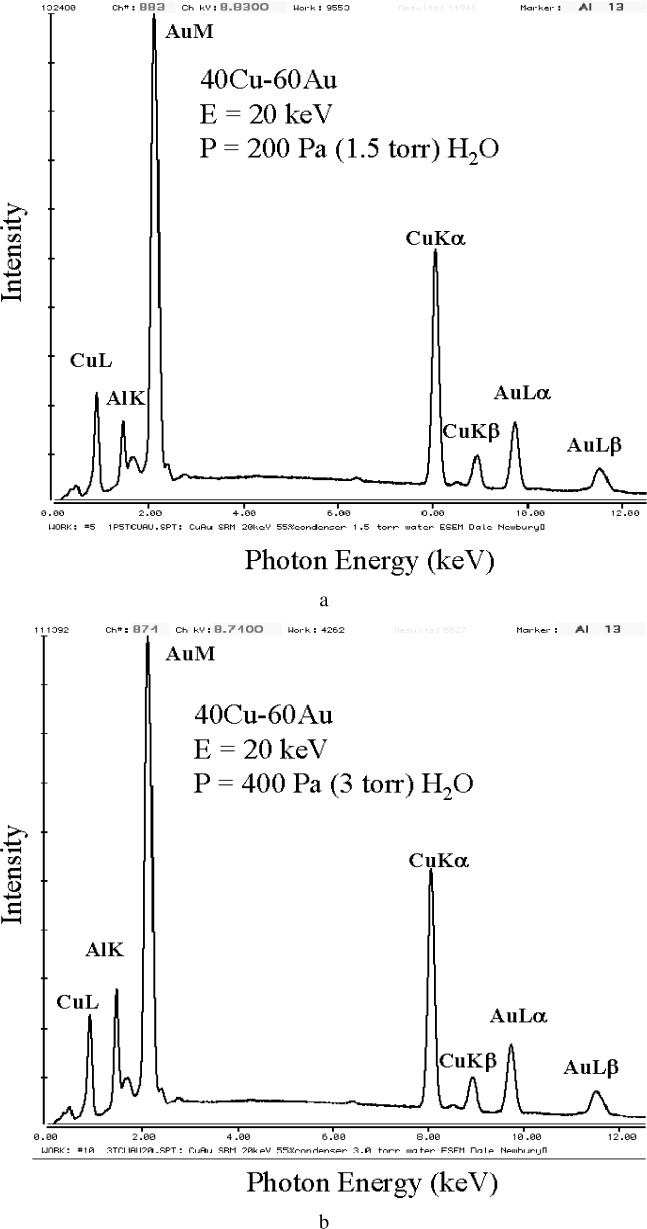

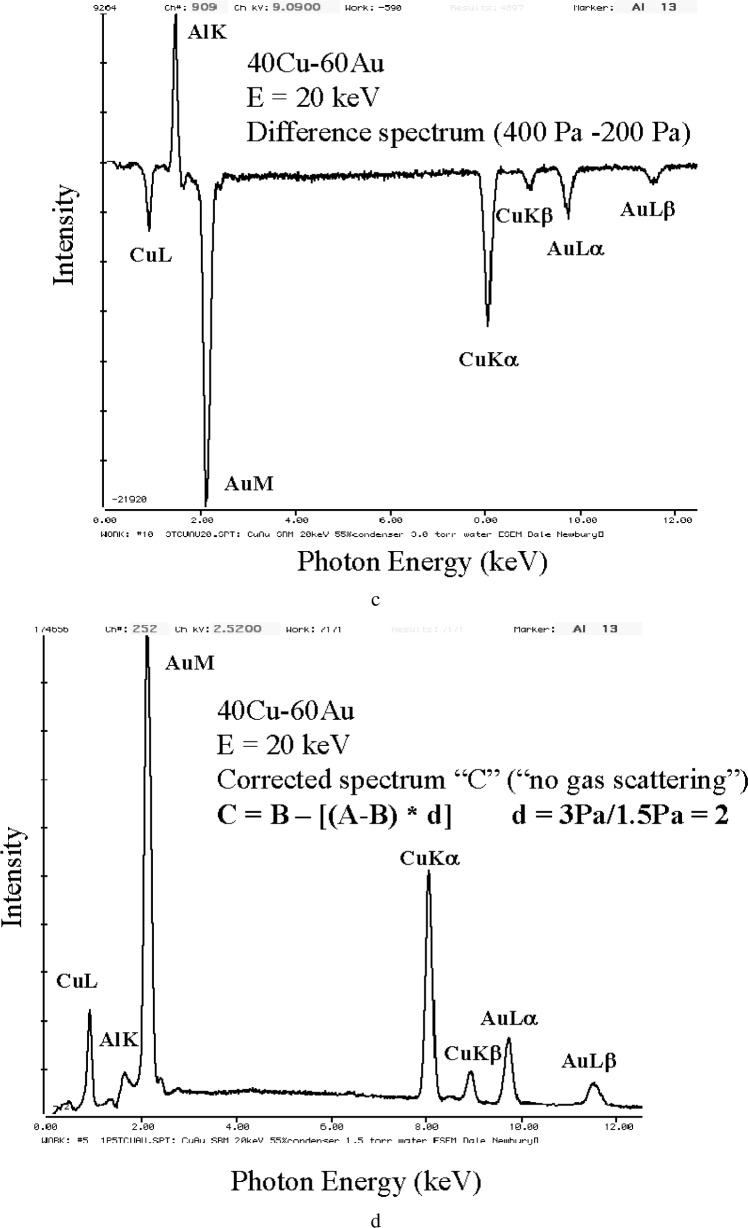
Application of the pressure shift method [8]. Correction of skirt contributions to the x-ray spectrum using data from Fig. 4 experiment: (a) 40Cu-60Au in Al mount, *E*_0_ = 20 keV, 200 Pa (1.5 torr) H_2_O; (b) 400 Pa (3 torr); (c) difference spectrum, 400 Pa to 200 Pa; (d) “no scattering” spectrum calculated using equation (2) with difference spectrum and a linear multiplier of 2.

This method is demonstrated in Fig. 7 using the spectra from the Cu-Au wire in Al block from Fig. 4. Spectra at 200 Pa (1.5 torr), Fig. 7 (a), and 400 Pa (3 torr), Fig. 7 (b), show the increase in AlK with pressure. These spectra are used to form the difference spectrum, Fig. 7 (c). Using Eq. (2) and a scaling factor derived from the ratio of the pressures results in the “corrected to no scattering” spectrum shown in Fig. 7 (d). Examining this spectrum, we note the complete elimination of the spurious Al-K peak, while the fine scale structure of the nearby AuMζ peak of the Au-M family is accurately retained, suggesting that, for at least this type of specimen which satisfies the Doehne-Bower criterion of no spectral change with skirt radius, major and minor peaks are well preserved [11].

**Fig. 8:**
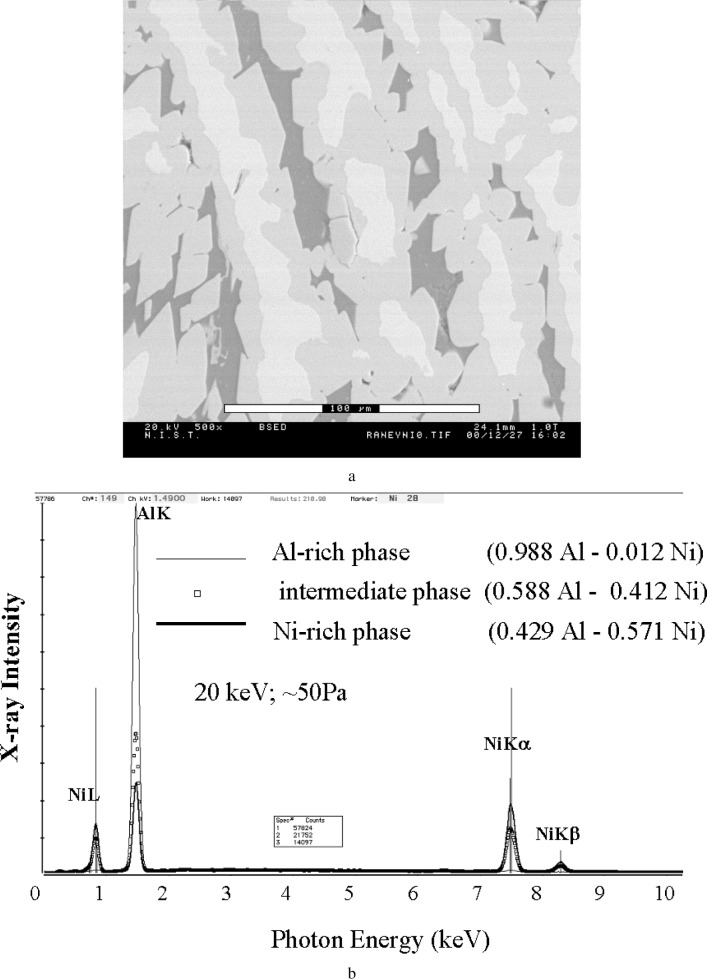

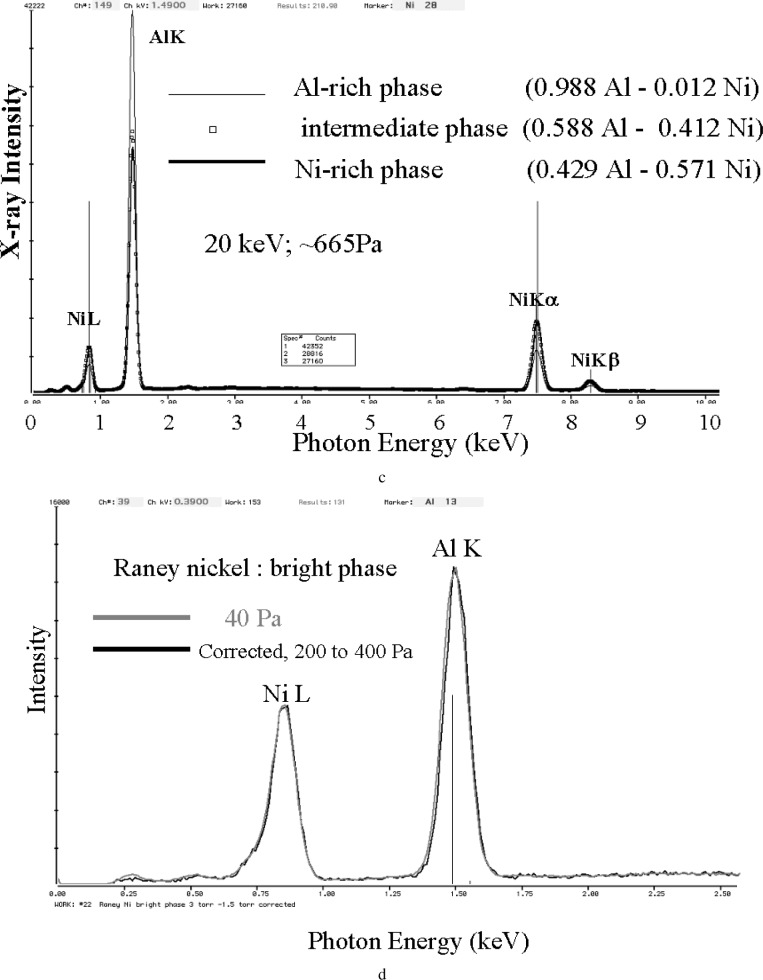

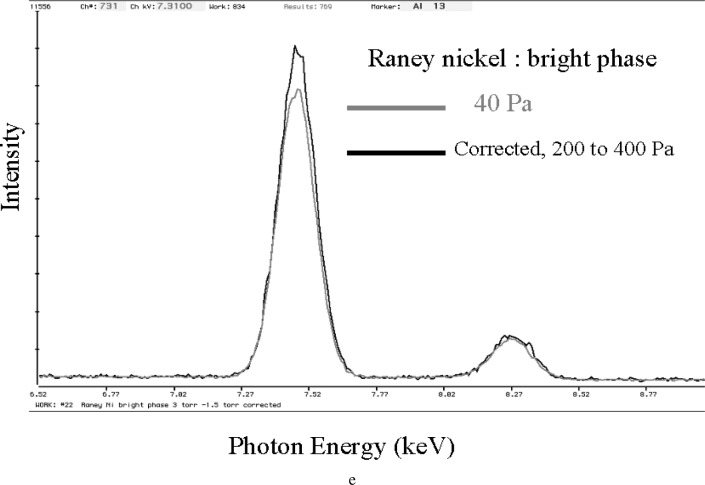
Application of the pressure shift method [8]. Correction of skirt contributions to the x-ray spectrum to a complex microstructure (Raney nickel). (a) Backscattered electron SEM image showing phase distribution; three distinct phases can be recognized. (b) EDS spectra observed on the three phases with a pressure of 50 Pa at a beam energy of 20 keV; (c) EDS spectra observed on the three phases with a pressure of 665 Pa at a beam energy of 20 keV; (d) Pressure corrected spectrum for the high—Ni phase showing the NiL and AlK peaks; (e) Pressure corrected spectrum for the high—Ni phase showing the NiKα and NiKβ peaks.

Figure 8 (a) shows a much more challenging analysis situation, a Raney nickel alloy microstructure with fine scale features having dimensions of approximately 10 µm to 20 µm, including three distinct phases with differing Al-Ni compositions, labeled “D” (dark, high aluminum), “I” (intermediate) and “B” (bright, high nickel). These three phases can be readily distinguished in the Si-EDS spectra taken at base pressure, Fig. 8 (b). When the pressure is increased to 665 Pa, Fig. 8 (c), the I and B phases can no longer be readily distinguished with the NiK x-ray peak, while the situation for the AlK peak is nearly as difficult. Table 2 shows the results of the pressure-variation correction procedure for a series of spectra measured on the highest average *Z* phase in Raney nickel (bright phase). The raw intensity data show the deviations observed from the “no scattering” situation as a function of pressure. As the pressure increases, the apparent phase composition deviates significantly from the “ideal” value found at low pressure. The results of corrections calculated with various choices of the pressure are also presented in Table 2. A “zero scattering” spectrum calculated with the spectra measured at 200 Pa and 400 Pa has a small error for AlK, but a larger error for NiK than either of the raw spectra. When the 400 Pa and 800 Pa spectra are used to calculate the “zero scattering” spectrum, the errors are similar in magnitude but the larger error is now found for the AlK peak. Finally, when the pressure range used to calculate the “zero scattering” spectrum is expanded to 200 Pa to 800 Pa, very large errors in both the AlK and NiK are found. This complex behavior most likely arises because of the complex arrangement and relative sizes of the phases of other composition that surround the particular location used for this measurement series. The gas scattering skirt situation for this alloy differs sharply from the circular symmetric geometry of the wire/disc experiment shown in Fig. 2. The magnitude of the errors suggests that relatively small pressure changes should be used to make corrections to determine the “zero scattering” spectrum.

### 3.3 Intercepting the Unscattered Beam

Bilde-Sorenson and Appel have described a method to estimate the contribution of the skirt based upon comparing two measurements, the first with the primary beam and the skirt striking the specimen and the second after intercepting the primary beam with a beam stop [9,10]. In one version, the beam stop consisted of a fine wire composed of an element that was not present in the specimen on interest. The second spectrum with the fine wire in place consists of contributions from the wire material and the skirt contribution. The peak(s) of the wire element are stripped from the beam stop spectrum, and what remains is assumed to be the skirt contribution. This skirt spectrum is then subtracted from the original spectrum to yield the spectrum due to the unscattered beam alone. In another variation of this technique, a foil of a unique element not contained in the specimen is used to cover the half of the specimen. The first spectrum is taken with the beam placed on the specimen near the foil, giving a composite spectrum consisting of the location on the specimen plus the skirt. The second spectrum is taken with the beam is placed on the foil close to the bare specimen, which yields the composite spectrum of the foil element plus the skirt contribution on the specimen. Again, the foil peak(s) is stripped off, and the remaining spectrum is considered to be the skirt contribution, which can then be subtracted from the first spectrum. This second procedure is especially suited to line scans made parallel to the edge of the foil. In practical applications, the foil is easier to accommodate than the wire, since this foil can be placed on the specimen externally relative to features of interest and no further manipulation is needed within the VPSEM-ESEM. The beam stop method involves the insertion of the fine wire above the specimen during operation, which requires a micromanipulator, and if a gaseous secondary electron detector (GSED) is in use, the conducting wire can interfere with the collection field of the GSED.

### 3.4 Bremsstrahlung Normalization Method

Griffin and Nockolds have described a method for quantitative x-ray microanalysis in the VPSEM-ESEM that makes use of an internal normalization based upon the use of a window of x-ray continuum [12]. Strictly, this method does not attempt to improve the resolution of the analysis by separating the skirt component from that of the direct beam, but rather seeks to re-establish the quantitative relationship between the intensity measured on a large area standard and on an unknown when gas scattering renders invalid the usual measures of beam current necessary for accurate dose normalization. A linear relation was observed between the mean atomic number of a target and the background counts in a specified high photon energy window. Monitoring the continuum intensity in this window provided an accurate correction for beam intensity variations of up to 50 % from a calibrated reference value in a conventional SEM. In the VPSEM-ESEM measurements, loss of intensity due to gas scattering outside of the standard would be manifested as a decrease in the intensity measured in the reference continuum window compared to the value expected in a conventional vacuum measurement without gas scattering. The authors noted that this correction resulted in VPSEM-ESEM quantitative measurements of similar accuracy as that achieved in conventional practice for silicate mineral samples. The principal constraint on this method is the requirement that as gas scattering occurs, it must simply reduce the x-ray intensity measured on the unknown and not contribute new characteristic x rays from material of different composition where the skirt impinges. As such, it is best suited to a specimen consisting of well isolated particles on a simple carbon background, as described below.

### 3.5 X-Ray Focusing Optics

**Fig. 9:**
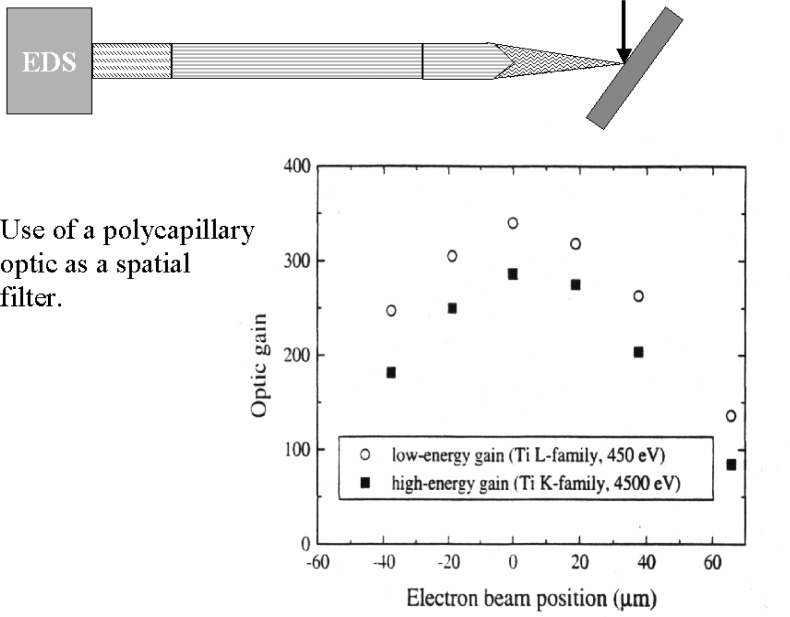
Use of a polycapillary x-ray optic (shown schematically) to serve as a spatial filter to restrict the acceptance area for x-ray collection. The plot shows the fall in intensity as a function of source position [14].

X rays can be efficiently focused by means of tapered polycapillary optics [13]. X rays approaching a surface below a certain critical angle are reflected with high efficiency. The critical angle is dependent on photon energy and material and is generally less than 0.01 rad. A capillary provides a rotationally symmetric reflector that can propagate x rays along its length through multiple low angle reflections. By gradually tapering the capillary, x rays can be made to follow a converging or diverging path. To maximize efficiency, the solid angle must contain as much reflecting surface as possible with as little solid glass, which acts to absorb the x rays. By bundling thin walled capillaries into polycapillaries, the amount of reflecting surface is maximized, and efficient optical components can be made. Figure 9 shows results of Wollman et al. on the focussing properties of one side of a double tapered polycapillary in which the electron excited source of x rays from titanium metal is moved in the plane perpendicular to the optic axis [14]. A sharp focus function is observed, such that the transmission is reduced by 60 % with approximately a 60 µm movement of the source off the maximum transmission point. Moreover, these measurements show similar behavior for photons spanning a broad energy range from Ti-L at 450 eV to Ti-K at 4500 eV. The absolute efficiency is energy dependent, with efficiency increasing for lower photon energies due to an increasing critical angle. The polycapillary optic can thus act as a spatial filter on a distributed x-ray source, such as that which exists in the VPSEM-ESEM due to the gas scattering skirt so as to exclude the remotely produced x rays while efficiently collecting x rays produced by the focused probe. In practice, x-ray mapping could be used to establish the position of the maximum transmission spot relative to the specimen position to maximize the efficiency. Such an optic would have the additional benefit of increasing the solid angle of collection of the EDS relative to an ordinary detector of the same size placed at the same distance. The spectrum measured with the optic would suffer the effects of the energy dependent transmission of the optic. Measurements of the x-ray continuum with and without the optic could be used to accurately establish the transmission function.

## 4. X-Ray Microanalysis in VPSEM-ESEM: Influence of the Specimen Configuration

### 4.1 The Analytical Blank

For certain specimen types, such as particles, the degree of success that can be achieved with x-ray microanalysis in the VPSEM-ESEM depends upon the configuration of the specimen. The central question is the validity of the measured EDS x-ray spectrum. When the focused beam is placed on the particle of interest, is the measured x-ray spectrum representative of the particle constituents? At what equivalent concentration level is the particle spectrum compromised by the remote scattering of beam electrons into the skirt? Can major (C > 0.1), minor (0.01 ≤ C ≤ 0.1), or trace constituents (C < 0.01) be trusted? Can the constituents, especially at minor and trace levels, be confidently assigned to the beam analysis position or must the analyst inevitably accept the much broader sampling of the beam/skirt combination? In the following discussion, progressively more complicated samples will be considered.

Underlying all measurements is the critical concept of the “analytical blank,” which is the irreducible background level of each constituent of interest contributed by all materials present except for the specimen itself. Determining and minimizing the analytical blank is especially important to achieve robust x-ray microanalysis in the VPSEM-ESEM.

#### 4.1.1 The Analytical Blank for Particle Analysis: Conventional SEM

The “analytical blank” for particle analysis by conventional SEM microanalysis is the spectrum of the specimen support substrate exposed to all stages of the specimen preparation procedure, including transfers in the laboratory environment, but without the specimen itself applied to the substrate. The spectrum obtained from the blank thus contains x-ray peaks arising from the substrate and adhesion materials as well as anything added as a result of specimen preparation and inadvertent contamination.

#### 4.1.2 The Analytical Blank for Particle Analysis: VPSEM-ESEM

**Fig. 10:**
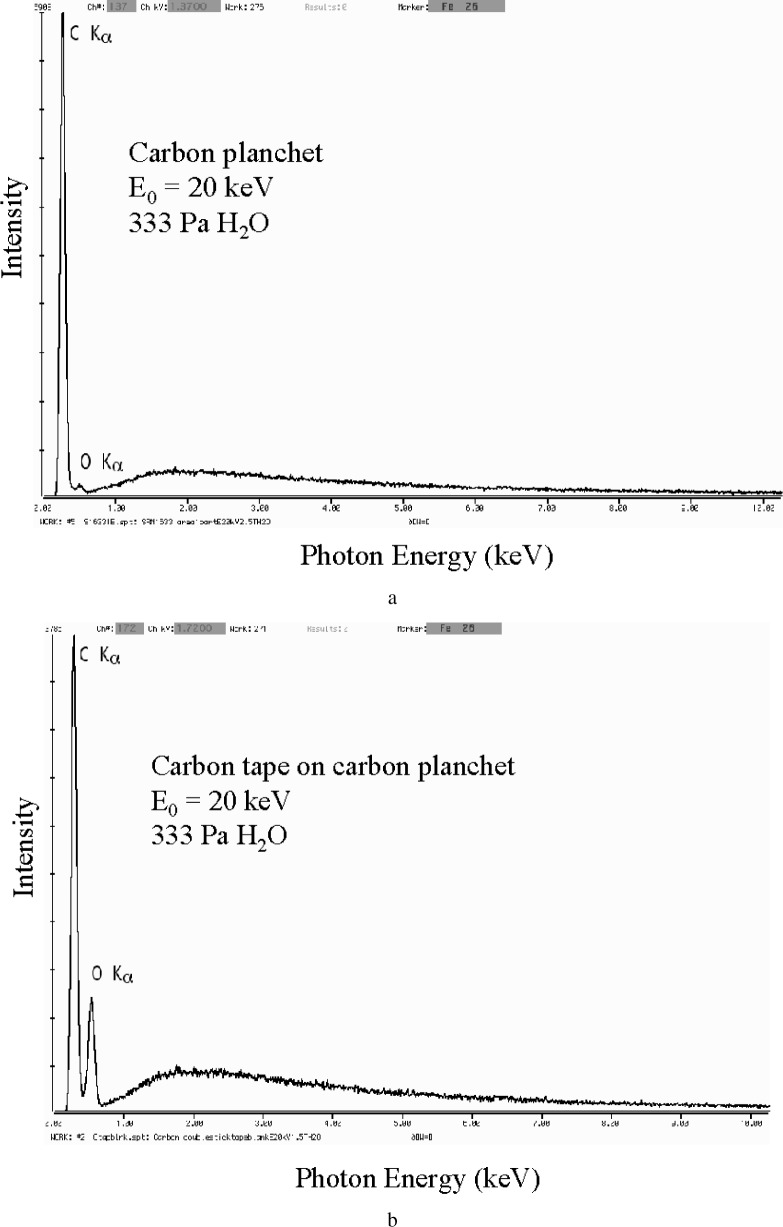
Analytical blank: (a) High purity carbon substrate; 20 keV beam; 200 Pa (1.5 torr) H_2_O; (b) Double-sided adhesive carbon tape on high purity carbon substrate; 20 keV beam; 200 Pa (1.5 torr) H_2_O.

For VPSEM-ESEM analysis, the analytical blank is more complicated and consists of two levels, the preparation blank and the operational blank. The preparation analytical blank is identical to the conventional blank, except that the substrate must be measured in the VPSEM-ESEM under identical conditions of gas species, pressure, and gas path length as will be used for the unknowns. Thus, gas scattering effects, including excitation of x rays from the environmental gas as well as the skirt electrons striking various materials, are included with the contributions from the direct beam striking the substrate and interacting with the environmental gas. Figure 10 (a) shows the Si-EDS spectrum of a preparation blank of a carbon planchet, in which a major carbon peak is observed with a small oxygen peak arising from the water vapor used as the environmental gas (333 Pa with a beam gas path length of 3 mm and a specimen to Si-EDS distance of 15 mm). Figure 10 (b) shows the spectrum of a preparation blank of carbon tape (with adhesive on both surfaces) mounted on a similar carbon planchet measured under the same conditions. A major carbon peak is again observed but with a substantially higher oxygen peak compared to Fig. 10 (a), with the additional oxygen arising from the adhesive and polymer tape substrate. Thus, this particular preparation is compromised with regard to the measurement of carbon at major constituent levels and oxygen at least to the minor constituent level by the constituents of the mounting materials.

**Fig. 11:**
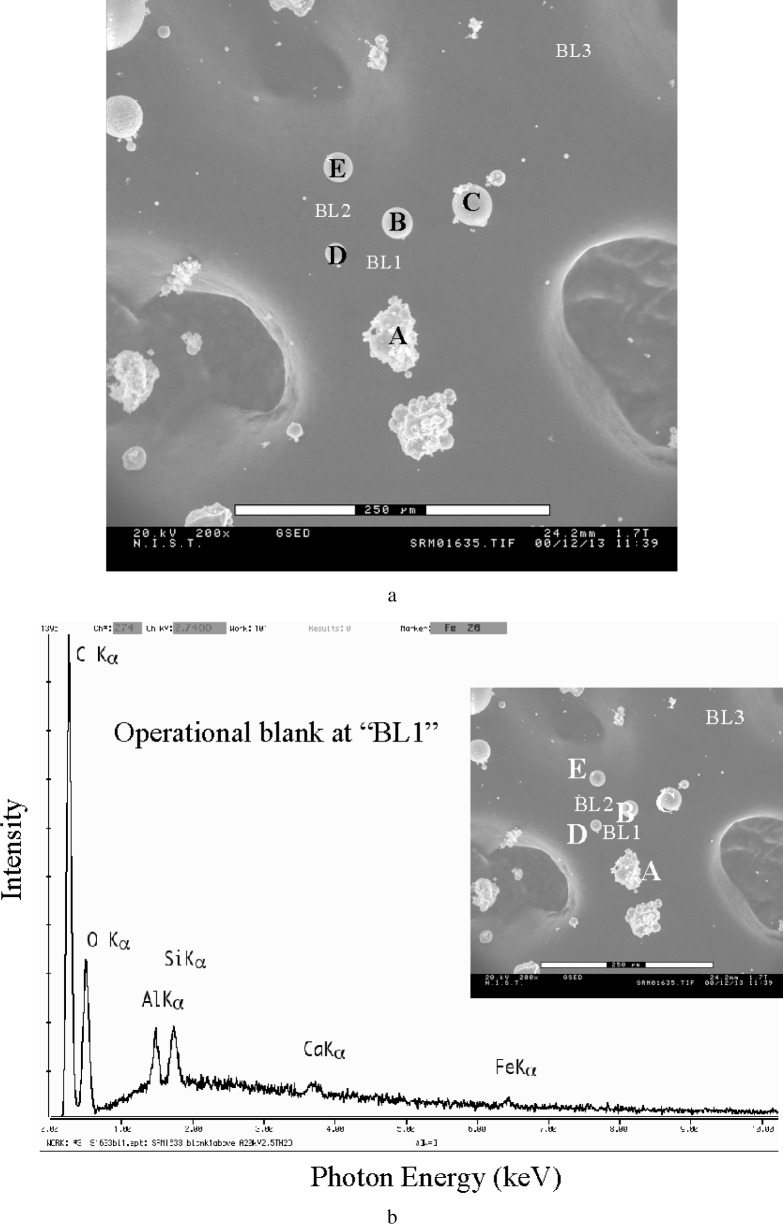

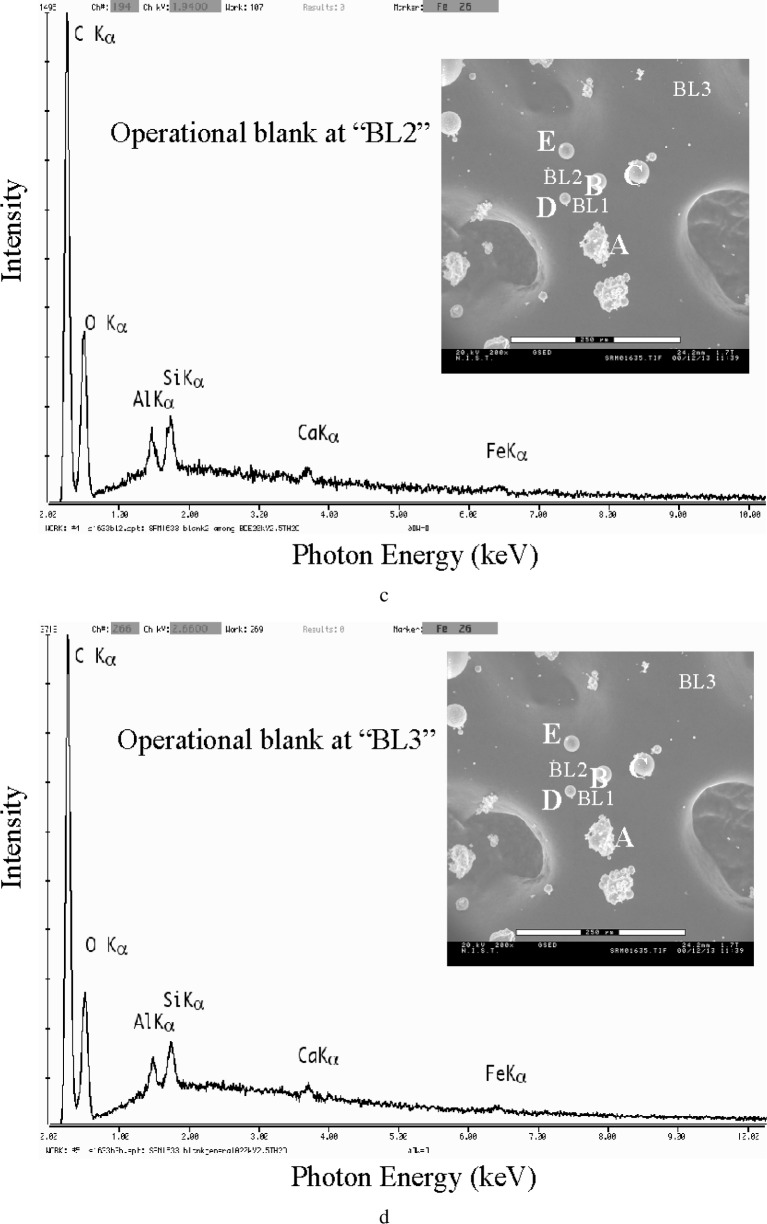

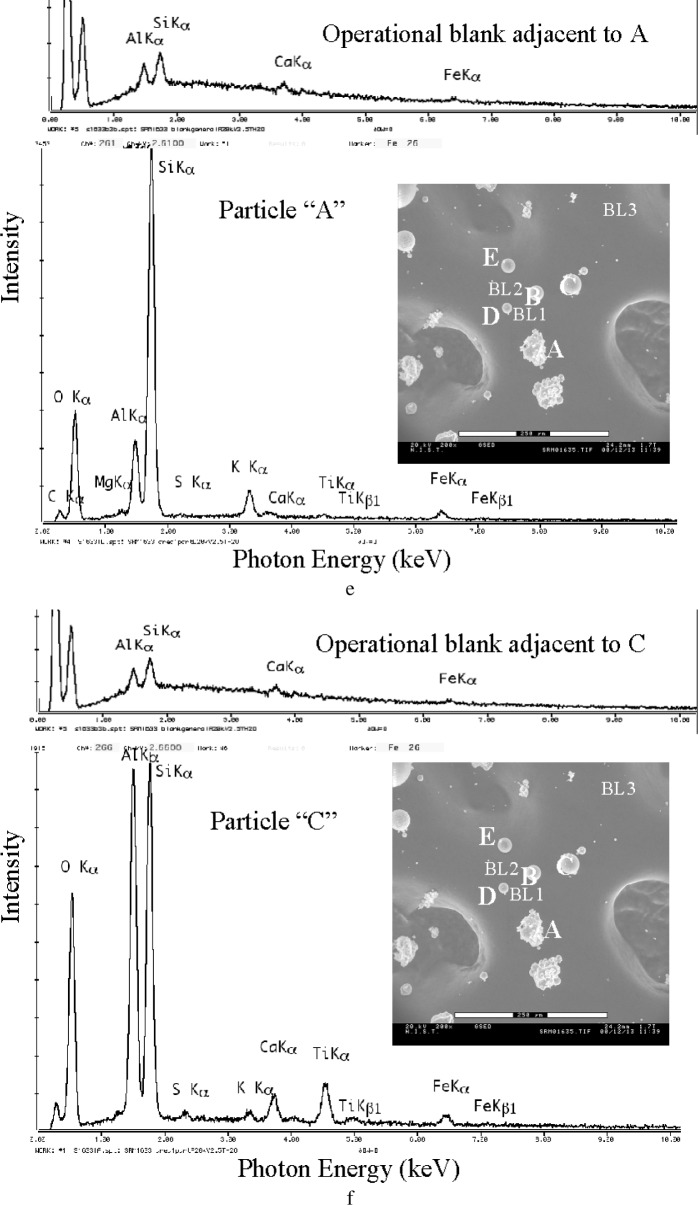

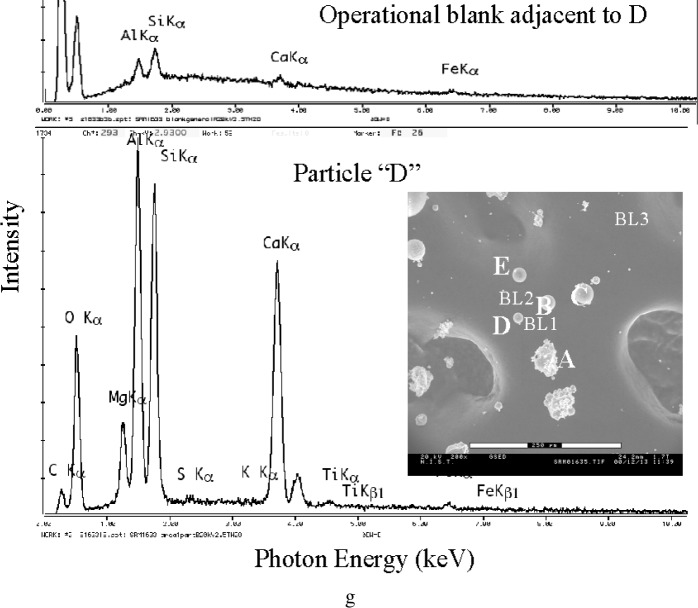
Particles of NIST SRM 1633 (Trace metals in fly ash): (a) micrograph of particles dispersed on double-sized adhesive attached to carbon planchet; (b) operational blank measured at location “BL1” in Fig. 5 (a); (c) operational blank measured at location “BL2” in Fig. 5 (a); (d) operational blank measured at location “BL3” in Fig. 5 (a); (e) particle spectrum measured with beam centered on particle “A” compared to operational blank; (f) particle spectrum measured with beam centered on particle “C” compared to operational blank; (g) particle spectrum measured with beam centered on particle “D” compared to operational blank.

The operational blank for VPSEM-ESEM considers the additional contributions that arise from the actual environment of the prepared sample, for example, from the particles surrounding the particle of interest in a dispersion deposited on a substrate. Figure 11 (a) shows a dispersion of particles of NIST SRM 1633 (Trace metals in fly ash) deposited on double-sided adhesive carbon tape, the preparation blank for which is shown in Fig. 10 (b) [5]. Figure 11 (b) shows an example of the operational analytical blank for this array of particles, as measured at the location marked in Fig. 11 (a). The consistency of the operational blank can be estimated by moving the beam off the particle of interest to a series of nearby locations, e.g., “BL1”, “BL2”, etc. marked in Fig. 11 (a). When this is done, the measured peak intensities represent the contributions from the skirt striking the substrate and other nearby particles. (Note that even with this careful protocol, the determination of the operational blank is necessarily imperfect because it does not consider the contribution due to electrons from the focussed beam that scatter off the particle of interest onto surrounding particles.) The operational blank can be expected to vary with beam position and with the exact arrangement of particles, and thus it should be measured at several locations to determine its variability. In this case, the operational blank spectra measured at “BL1” [Fig. 11 (b)], “BL2” [Fig. 11 (c)], and further away at “BL3” [Fig. 11 (d)] are very similar, although this does not have to be the case. The low but distinct levels of aluminum, silicon, calcium, and iron are the result of the skirt electrons striking other particles in the neighborhood, while carbon and at least some of the oxygen arise from the substrate and tape, as determined from the preparation blank. After determining the operational blank, a representative spectrum should be compared to each measured particle spectrum. Examples are shown for several of the particles in Fig. 11 (a) in Figs. 11 (e), (f), and (g). Taking into account the peak heights observed when the beam was placed on pure element standards, the operational blank indicated that several elements were compromised in particle measurements: carbon as a major constituent; oxygen, aluminum and silicon as minor constituents; and calcium and iron as trace constituents. The situation might change in another area on the same specimen where particles are more widely dispersed, or different species are present, so the operational blank must be continually updated for each local region.

### 4.2 Isolated Particles

#### 4.2.1 Particles Dispersed on Carbon-Containing Substrates

Particles are often collected on non-conducting filter media such as paper and plastics. Such filters may be impossible to examine directly in the conventional SEM, even with heavy coating, because the extreme topography of the filter leads to inadequate coating coverage and subsequent charging. In such cases, this necessitates removal of the substrate and/or transfer of the particles. However, because microscopic particles are often easily modified morphologically and chemically when exposed to the solvents needed to remove the filter medium, such removal and transfer may not be possible without compromising the information that is sought. Situations that require examination of particles as collected on the filter medium represent an ideal opportunity for LVSEM-ESEM microscopy and microanalysis.

The optimum sample configuration to achieve nearly uncompromised x-ray microanalysis in LVSEM-ESEM is that of widely dispersed particles. Wide dispersal minimizes contributions to the operational blank from nearby particles and decreases its variability. The particles are deposited on a simple substrate, most typically high purity carbon, at a loading density such that the particles are spaced by at least 10 to 100 times their largest lateral dimension. With such a wide dispersion, the spectrum of an individual particle should be uncompromised for major and minor constituents, with the exception of those elements noted in the preparation blank that arise from the substrate and the environmental gas. To determine the validity of trace element peaks, the analyst must be prepared to carefully examine the operational blank associated with each particle. Carlton tested this situation with artificial constellations of particles (dimensions from 100 µm to 400 µm) [15]. For example, with a beam energy of 20 keV, a chamber pressure of 599 Pa (4.5 torr) of water vapor, and particles (approximately 10 µm dimensions) separated by approximately 3.5 mm, the contribution of the remote particles [pure copper, glass (of unspecified composition), and the mineral cassitterite] to the central spectrum of a titanium particle occurred at the level of Cu-K/Ti-K = 0.0014 and Sn-L/Ti-K = 0.00029, which are just at the threshold of trace detection for EDS performed with practical dose conditions.

#### 4.2.2 Reducing the Background: Use of Thin Foil Substrates

**Fig. 12:**
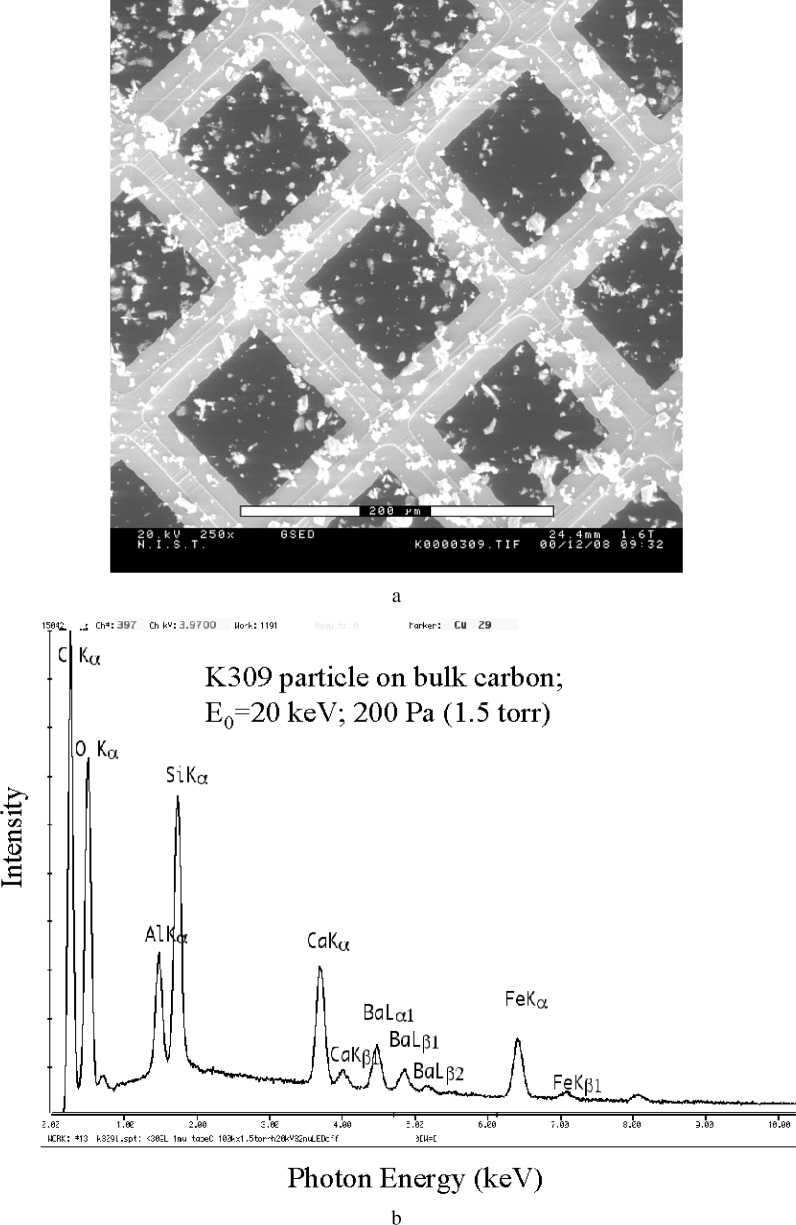

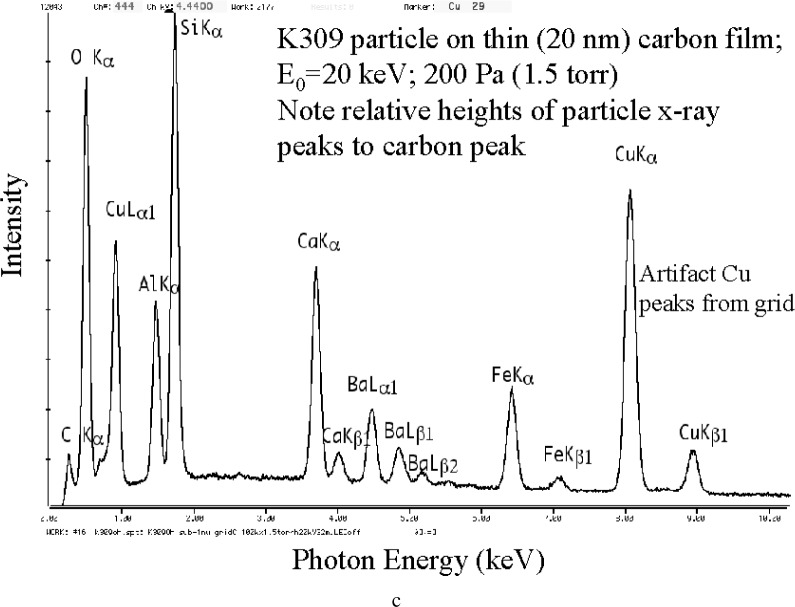
Particles of NIST glass K309 deposited on thin (~20 nm) carbon film supported on a copper grid: (a) low magnification view of particle dispersion showing 80 µm (on edge) windows. Spectra of individual, micrometer-sized particles of NIST glass K309, beam energy 20 keV; pressure: (b) on bulk carbon adhesive tape, note the large carbon peak; (c) on carbon thin film (≈ 20 nm) carried on copper grid. Note artifact peaks of Cu-L and CuKα, CuKβ peaks from scattering onto grid, but near absence of carbon peak.

As the size of the particle is reduced, the relative proportions of the spectrum contributed by the direct beam striking the particle and by the skirt striking the bare substrate and surrounding particles change. While the skirt contribution remains constant, the x-ray intensity generated by the direct beam on the particle decreases with particle size, especially for particles below approximately 1 µm in thickness (i.e., the dimension along the beam). For sufficiently small particles, the remote skirt spectrum from the bulk carbon substrate will eventually dominate the particle spectrum. After the operational parameters of beam energy, environmental gas species, gas pressure, and beam path length have been chosen, the remaining variable to reduce the skirt contribution to the composite spectrum is to modify the substrate itself. A low mass thickness substrate can be obtained with a free standing carbon film (20 nm nominal thickness) supported on a electron microscope grid (e.g., copper, nickel or carbon). Such a thin carbon film is surprisingly strong, and particles can be deposited on the film by various methods, including by drying particle-loaded liquid droplets. This grid with the particle deposit is then placed over a deep (at least 5 mm) blind hole in a carbon block so that electrons passing through the film are likely to be absorbed by the substrate and not backscattered to strike the specimen or grid again. Figure 12 (a) shows such a preparation of NIST K309 glass particles on a carbon film (20 nm nominal thickness) supported on a copper grid with 80 µm square openings. Figures 12 (b) and (c) show a comparison of EDS spectra from similar, micrometer-sized K309 particles on a bulk carbon tape substrate as compared to carbon thin film substrate. Relative to the aluminum and silicon peaks from the glass, the spectrum from the film on grid preparation shows carbon reduced by a factor of at least 10 compared to the spectrum measured on the bulk carbon tape substrate, where carbon is the highest peak. Note that the spectral peak-to-background is higher for the thin film support. At Si, the *P*/*B* is approximately twice as high, and therefore the measured spectrum consists of a larger contribution from the particle relative to the substrate compared to the equivalent case with a bulk carbon substrate. For sub-micrometer particles, the improvement in *P*/*B* would be even greater. Note that the particle spectrum from the thin film also contains an additional artifact, the x rays emitted when the skirt electrons strike the support grid, which is copper in this case. To avoid this artifact, other metallic and nonmetallic support grids are available, including other metals (Ni, Al, stainless steel), carbon and nylon.

#### 4.2.3 Alternatives to Carbon Substrates

If carbon is of interest in the particles, other high purity elemental substrates such as aluminum foil or silicon wafers are readily available. Often Al and Si are of interest themselves, so these materials may not be satisfactory choices for the substrate. As an alternative, high purity beryllium would be of particular interest as a substrate, since its characteristic x-ray is of such low energy (110 eV) that it is not detectable by most EDS systems. Unfortunately, beryllium in the form of beryllium oxide is highly toxic, and this fact greatly constrains its use, especially if the planchet surface must be polished produce a flat surface with the inevitable possibility of contamination of the laboratory. As an alternative, elemental boron and its oxide are not significantly toxic, although its x-ray peak at 185 eV is detectable with high performance EDS systems. Boron is extremely hard, and a highly polished surface can be produced with an appropriate polishing protocol [16].

### 4.3 Fibers

**Fig. 13:**
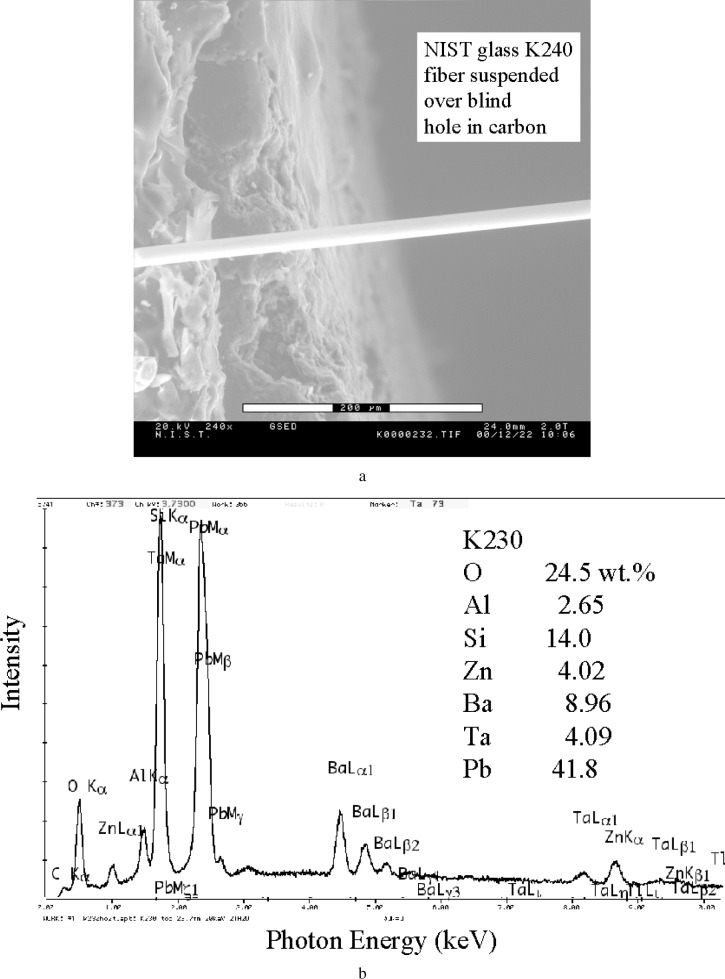

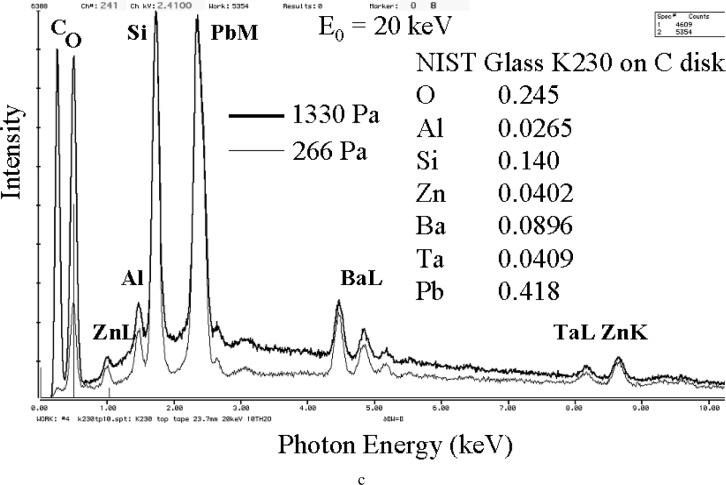
VP-ESEM image of a fiber of NBS glass 230 suspended over a hole in a carbon block: (a) position shown where the fiber passes over the edge of the hole; (b) the x-ray spectrum of a 16 µm diameter fiber obtained at 266 Pa (2 torr, H_2_O) with the 20 keV beam placed in the center of the hole; (c) spectrum obtained where the same fiber is attached to the carbon tape on the bulk carbon.

If the specimen is in the form of an individual fiber, it is again possible to improve the quality of the x-ray spectrum by reducing or even completely eliminating the skirt contribution from the substrate. By placing the fiber over a large diameter, deep, blind hole in a carbon block, the skirt electrons have nothing with which to interact within the solid angle of acceptance of the EDS and their contribution is effectively eliminated except for the environmental gas itself. An example of this approach is seen in the images in Fig. 13 (a), where a fiber of NIST glass K230 (O = 0.0245 mass fraction; Al = 0.0265 mass fraction; Si = 0.0140 mass fraction; Zn = 0.0402; Ba = 0.0896 mass fraction; Ta = 0.0409 mass fraction; Pb = 0.0418 mass fraction), is suspended over a deep, 3 mm diameter hole while attached on either end to a pad of carbon tape. The x-ray spectrum of a 16 µm diameter fiber obtained at 266 Pa (2 torr, H_2_O) with the beam placed in the center of the hole is shown in Fig. 13 (b), while the spectrum obtained where the same fiber is attached to the carbon tape is shown in Fig. 13 (c), where the carbon peak from the skirt is prominent. Note that the carbon peak is almost completely eliminated in the 266 pA (2 torr) spectrum recorded in the center of the hole. Thus carbon-bearing fibers could be successfully characterized by x-ray spectrometry with this specimen mounting procedure, which could be improved even further through the use of a support that did not contain carbon, e.g., by supporting the fiber across a hole in an aluminum disk, or some other metal not of particular interest.

**Fig. 14:**
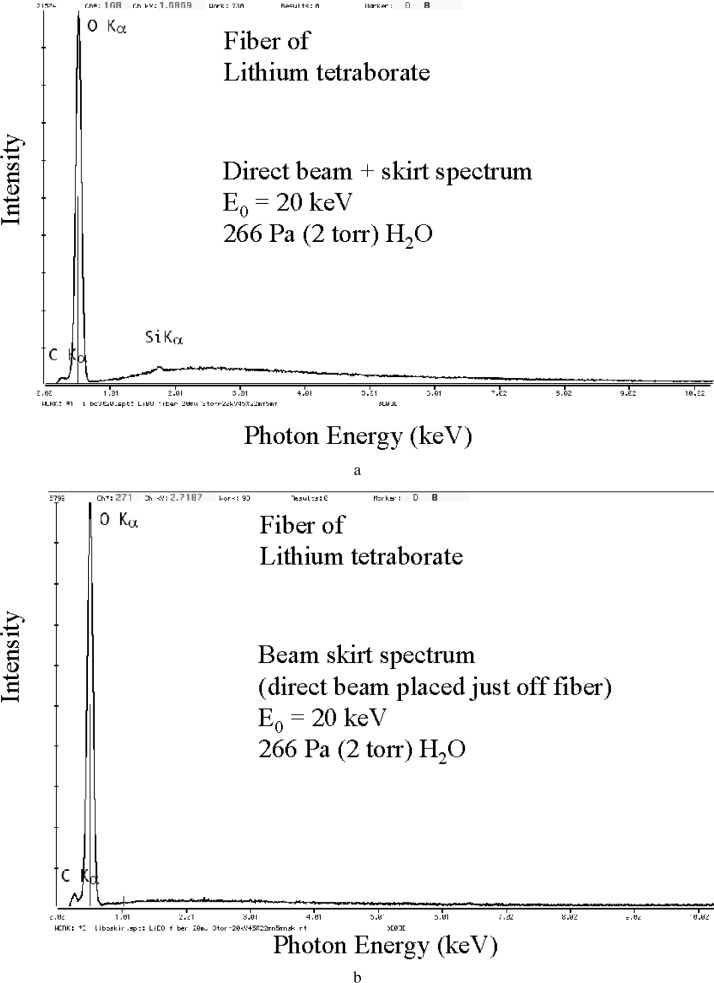
Use of a fiber of lithium tetraborate glass as a specimen support: (a) EDS spectrum with direct beam on fiber; 400 Pa (3 torr); 20 keV; (b) EDS spectrum contributed by skirt, a factor of 3 lower.

**Fig. 15:**
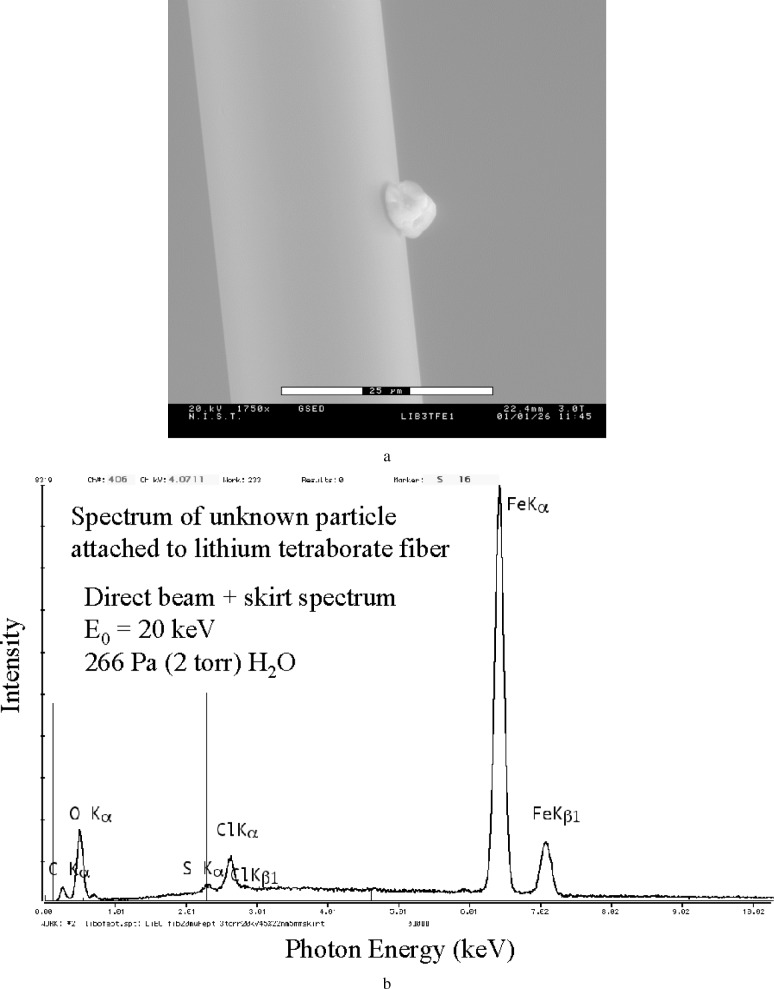
(a) ESEM image of an unknown particle attached to lithium tetraborate glass fiber; (b) EDS spectrum with direct beam on particle; 400 Pa (3 torr); 20 keV.

A further variant of this fiber technique can be used to obtain high quality spectra of particles. A small diameter fiber with a thin layer of adhesive can be used to mount particles for suspension over a hole. While the material of the fiber will contribute to the spectrum, it may be possible to make this contribution insignificant to the particle spectrum with careful choice of the mounting fiber material. Lithium tetraborate glass fibers would be of particular use here, since the only significant spectral contribution would be from the oxygen component. Figure 14 (a) shows an example of a blank spectrum from a 20 µm diameter fiber of lithium tetraborate glass suspended over a 3 mm diameter hole in a carbon block. The only significant characteristic peak is that of oxygen. The corresponding skirt spectrum obtained with the beam placed just off the fiber is shown in Fig. 14 (b); this spectrum is virtually identical but the oxygen peak intensity is a factor of three less. Figure 15 (a) shows a 5 µm particle of unknown composition attached to this fiber. The EDS spectrum of this particle, Fig. 15 (b), reveals a large iron peak, and lower intensities for sulfur and chlorine. The particle may also contain oxygen, but the interference from the strong oxygen peak from the lithium tetraborate glass fiber precludes interpretation.

### 4.4 Bulk Specimen

#### 4.4.1 Homogeneous, Single Phase

Bulk specimens that are compositionally homogeneous over large regions and consist of a single phase can be analyzed in VPSEM-ESEM without significant difficulty. As long as the lateral specimen dimensions exceed the diameter of the skirt, as determined with Eq. (1) and/or with Monte Carlo simulation, then the effect of the gas scattering is simply to degrade the size of the probe, but in the absence of a microstructure, this spatial degradation is unimportant. Since the energy lost during gas scattering is not significant, all of the electrons in the broadened probe, including those in the skirt, are equivalent in terms of x-ray excitation. X rays from the environmental gas can contribute to the spectrum as the only significant artifact, and if the gas pressure is at the high end of the ESEM range (> 1500 Pa), absorption can occur, especially for low energy peaks. Operation in the VPSEM pressure range or the low portion of the ESEM pressure range should effectively eliminate both of these artifacts. Thus, except for the loss of spatial resolution, the analysis of large, homogeneous targets in the VPSEM-ESEM is essentially equivalent to microanalysis performed in the conventional high vacuum SEM but with a highly defocused beam.

Since the skirt electrons are energetically equivalent to the beam electrons, it should be possible to perform quantitative x-ray microanalysis in the VPSEM-ESEM for large, homogeneous targets. The most rigorous quantitative x-ray microanalysis procedure is based upon the measurement of standards (e.g., pure elements or stoichiometric compounds) under the same conditions (beam energy, known dose, and spectrometer efficiency) as the unknown followed by the calculation of matrix correction factors to convert x-ray intensity ratios for each x-ray peak (sample/standard) into concentration ratios [1]. In principle, standard intensities could be measured for the unscattered beam, while the gas-scattered beam with the same total current could be used for the large, homogeneous unknown. Alternatively, very large standards could be used to accommodate the skirt electrons, but this may be procedurally difficulty when the need is for a large suite of standards.

In actual practice, it is very difficult in the VPSEM-ESEM to establish this measurement equivalence between the unknown and the standards. The large lateral spread of the gas-scattered beam results in the possibility that some portion of the skirt electrons may strike the unknown in areas where the efficiency of the EDS drops off. This situation is further complicated by the use of a support grid for the ultrathin window in the modern EDS spectrometer. The detector window efficiency depends strongly on the position at which an x-ray strikes the window. If the x-ray path intersects the grid, the low energy photon efficiency drops off severely. When all of the x rays are effectively produced as a point source with lateral dimensions of only a micrometer, the illumination of the EDS window is well defined. When the x rays are produced over a large spatial area as in the case of the VPSEM-ESEM, then the illumination of the EDS window is much more complex and difficult to bring under measurement control. Moreover, if the region excited by the skirt extends outside the area of acceptance of the collimator of the EDS, then it is critical that the unknown and standard be located at the same position relative to the center of the beam of the VPSEM-ESEM and the central axis of the EDS. With sufficient care in this positioning and using bremsstrahlung to compensate for dose variations, Griffin and Nockolds have demonstrated satisfactory results in the quantitative analysis of large mineral specimens with ESEM-EDS [12].

As a result of this difficult measurement situation, “standardless” analysis, in which the necessary standard intensities are calculated theoretically or else estimated from remotely measured standards, is often the method of choice for the VPSEM-ESEM. It must be recognized, however, that “standardless” analysis procedures, even under optimal conditions in the conventional SEM, have been shown to produce much broader error distributions than those obtained with standards [17,18]. Before reporting to a customer the results of any “standardless analysis procedure” applied in a VPSEM/ESEM, it is strongly recommended that the analyst assess the accuracy of the standardless procedure by testing it against known multi-component homogeneous standards measured under conventional high vacuum conditions.

**Fig. 16:**
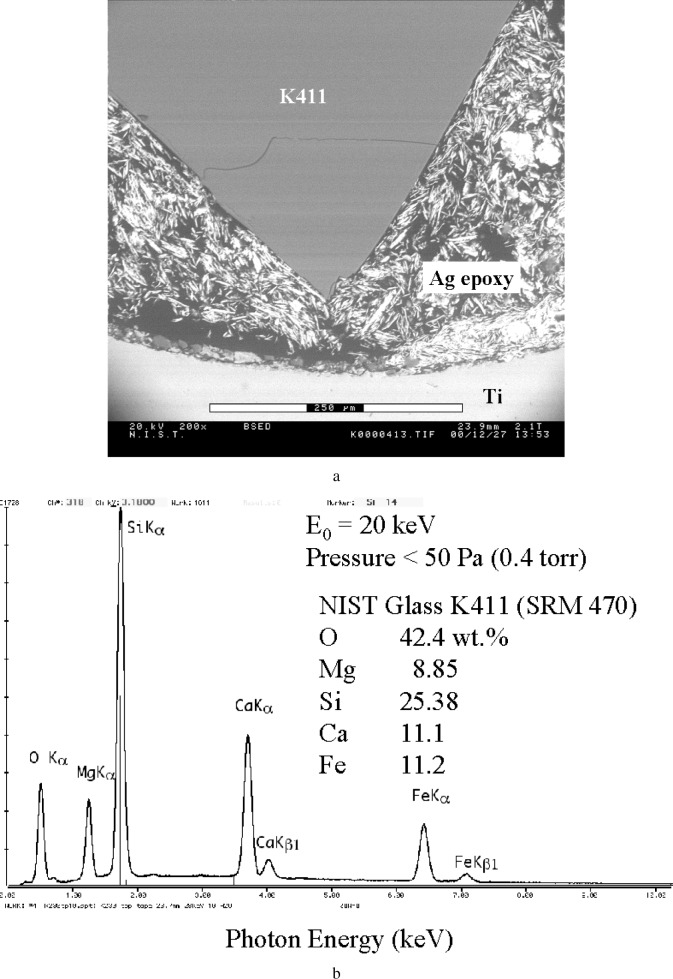

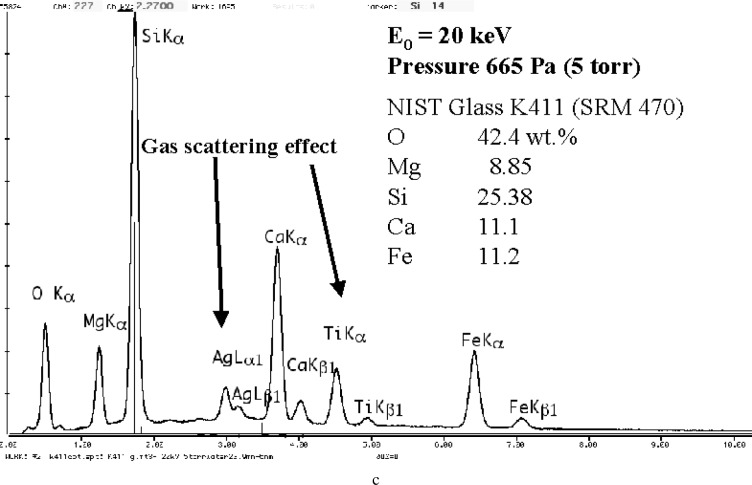
(a) VP-ESEM image of a polished block of NBS glass K411 (SRM) mounted in silver-loaded epoxy and held in a titanium block (b) EDS spectrum at low pressure (< 50 Pa, 0.4 torr) with no gas scattering showing the peaks expected (K-lines of O, Mg, Si, Ca, and Fe); (c) EDS spectrum at 665 Pa (5 torr) of water vapor showing additional peaks for silver (Ag-L) and titanium (Ti-K).

Figure 16 (a) shows an example of a homogeneous block of glass embedded in silver-loaded epoxy in a hole drilled into a block of titanium. Figure 16 (b) shows the EDS spectrum obtained with a pressure below 50 Pa (0.4 torr), in which the only significant peaks are those for the known constituents of the glass. When this spectrum was processed through the standardless analysis procedure embedded in the commercial analytical software package that supported the particular EDS spectrometer used, the results given in Table 3 were obtained. The relative errors are typical of the distribution observed in a detailed test of standardless analysis performed previously [18]. When the pressure was increased to 650 Pa (5 Torr), the spectrum included additional peaks arising from the silver-loaded epoxy and the titanium block. By instructing the quantitative analysis software to ignore the Ag-L and Ti-K peaks, the results listed in Table 3 were obtained, with similar but somewhat larger relative errors compared to the unscattered case. The increase in the error observed at the higher pressure may be due to increased uncertainty in the peak fitting introduced by the additional x-ray peaks contributed by the skirt. Since normalization is forced upon the results in standardless analysis, this has the effect of distributing the error over all components.

#### 4.4.2 Heterogeneous, Multiphase Microstructures

The most difficult analytical situation for the VPSEM-ESEM is the case of a heterogeneous microstructure with two or more phases, such as a discontinuous phase in a bulk matrix (e.g., an inclusion in a matrix), especially when the problem involves determining elemental constituents that are partitioned between the two phases. As usual, the effective beam footprint due to gas scattering acts to produce a composite spectrum from all of the phases. This effect is shown in Fig. 10. A three-phase microstructure, Fig. 10 (a), produces sharply different spectra when measured under pressure conditions (< 50 Pa), Figs. 10 (b), (c), (d), while with significant gas scattering at 650 Pa of water vapor, the spectra begin to converge, Figs. 10 (e), (f), and (g). The bright and intermediate phases, Figs. 10 (e) and (f), are virtually indistinguishable, while the dark phase, which actually contains a very low level of nickel, as seen in Fig. 10 (d) for the no gas scattering condition, appears to contain significant nickel due to gas scattering, Fig. 10 (g). The level of spectral differentiation that will be observed obviously depends very strongly on the exact compositional nature and scale of the of the microstructure relative to the gas scattering footprint. While a series of spectra measured as a function of pressure might make it possible to deduce the no-gas-scattering condition, it is clear that these measurements would have to be made with extreme care to maintain a consistent gas scattering situation.

## 5. X-Ray Mapping in the VP-ESEM

X-ray mapping provides powerful visual information on the spatial distribution of elemental constituents at micrometer lateral resolution and is one of the most widely applied qualitative analysis procedures. An x-ray map is created by assigning a gray level in the image storage/display according to the x-ray intensity measured at each pixel. Generally, the intensity is measured (and stored) on a 16 bit deep scale (maximum 65, 536 counts), but for display purposes the intensity range is scaled to 8 bits (0–255), with the maximum intensity set to white (level 255). In the conventional mapping procedure that is incorporated in most commercial software systems, no background or peak overlap corrections are applied, so that the intensity maps may be valid only for major constituents (concentrations above 0.1 mass fraction). For minor and trace level constituents, the continuum background forms a progressively larger fraction of the measured intensity as the concentration decreases, so that the atomic number dependence of the x-ray continuum eventually dominates the contrast in the x-ray map. With background and overlap corrections, mapping can be useful for minor and even trace constituents. A good quality assurance procedure in x-ray mapping is to record x-ray spectra of each phase that apparently contains minor and trace constituents to determine the validity of any apparent contrast between phases observed in maps. If the x-ray contrast between any pair of phases is valid, it should be confirmed by a proportional change in the relative peak heights in spectra measured from those phases. Invalid contrast situations may arise due to the atomic number dependence of the x-ray continuum, but this situation will be revealed in spectra taken in the regions in question.

**Fig. 17:**
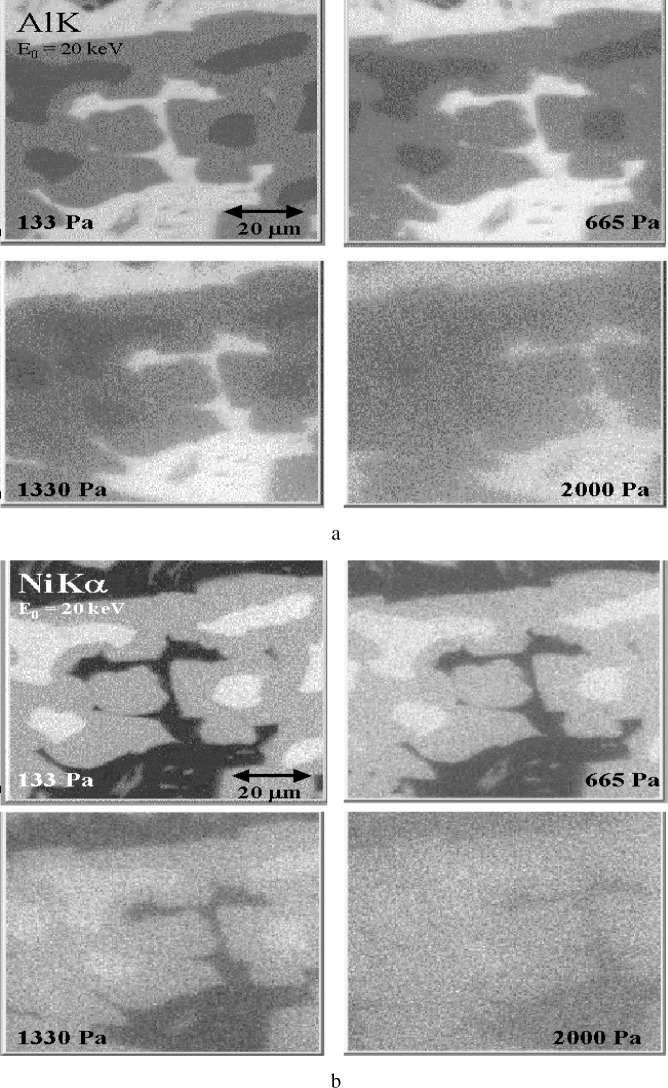
X-ray mapping in the VPSEM/ESEM: Raney nickel, *E*_0_ = 20 keV; all maps at 4 mm gas path; H_2_O at pressures of 133 Pa (1 Torr), 665 Pa (5 Torr), 1330 Pa (10 Torr), 2000 Pa (15 Torr); *E*_0_ = 20 keV; (a) Al x-ray maps; (b) Ni x-ray maps.

X-ray mapping is subject to the additional artifacts discussed throughout this chapter that are peculiar to the VPSEM-ESEM. The most serious is the action of gas scattering of the primary beam to degrade the effective spatial resolution through remote excitation of x rays. A particular consequence of gas scattering for x-ray mapping is the decrease in contrast between phases as the beam gas path length increases. This artifact is especially serious for bulk specimens where the remotely scattered electrons excite characteristic x rays from the same elemental species as in the mapped area. Figures 17 (a) and (b) show the results of x-ray mapping for the major Al and Ni constituents of Raney nickel alloy over a range of pressures. The maps obtained at the lowest pressure [133 Pa (1 torr) and a 4 mm gas path] showed three distinct major phases, sharply defined by the variation in both the Al and Ni intensities. Quantitative EDS analysis of these phases (performed in a conventional SEM with pure element standards and quantitative calculations with Desktop Spectrum Analyzer) yielded the results shown in Table 4, which indicate the degree of compositional contrast.) As the chamber pressure was progressively increased from 133 Pa (1 torr), 665 Pa (5 torr), 1330 Pa (10 torr), and finally 2000 Pa (15 torr), the x-ray maps showed a decrease in resolution and in the contrast between phases. The visibility of the intermediate and bright phases, which yield a concentration contrast of about 25 % from Table 4, decreased sharply and was only barely visible in the maps recorded at 1330 Pa (10 torr). At 2000 Pa (15 torr) this contrast was completely lost, and only two phases could be discerned.

## 6. Conclusions

X-ray microanalysis in the VPSEM and ESEM can be a useful tool to complement SEM imaging, but the analyst must recognize the inevitable limitations that result from gas scattering compared to the level of analytical performance achieved in a conventional high vacuum SEM. The impact of gas scattering on both qualitative and quantitative analysis generally increases as the concentration of a constituent of interest is lowered from major (*C* > 0.1 mass fraction), to minor (0.01 ≤ *C* ≤ 0.1), to trace (*C* < 0.01 mass fraction) levels. While it is usually possible to achieve useful results for major constituents, minor and trace constituents are likely to be severely compromised. To minimize the effects of gas scattering, the beam gas path length must be made as short as possible, consistent with accommodation of the EDS x-ray spectrometer. For certain classes of specimens, such as particles and fibers, the analyst can also seek to minimize the contributions of the background through the use of thin film supports (particles) and suspension over holes (fibers). Quantitative analysis of areas with micrometer dimensions is severely compromised, but methods have been developed to correct for the contributions of the electron scattering skirt by measuring spectra over a range of pressures. X-ray mapping under VPSEM-ESEM conditions suffers in terms of the minimum compositional contrast that can be detected, as well as in terms of degraded spatial resolution.

## Figures and Tables

**Table 1:** Characteristic x-ray transmission by the environmental gas (oxygen) (specimen to EDS window: 4 cm)

Element/X-ray	*I*/*I*_0_ (2500 Pa)	*I*/*I*_0_ (100 Pa)	*I*/*I*_0_ (10 Pa)
F K	0.194	0.940	0.994
NaK	0.572	0.979	0.998
AlK	0.805	0.992	0.9992
SiK	0.868	0.995	0.9995
S K	0.939	0.998	0.9998
ClK	0.957	0.998	0.9998
K K	0.986	0.999	0.9999
CaK	0.990	0.9996	0.9999

**Table 2:** Analysis of raney nickel high-*Z* phase with corrections applied for gas scattering

Pressure (Pa)	AlK	% Deviation ref.	NiK	% Deviation ref.
50	149826		153029	
200	169089	+13 %	155919	+1.9 %
400	192770	+29 %	140844	−8 %
800	212216	+42 %	121235	−21 %
Corr. (400–200)	145576	−3 %	170915	+12 %
Corr. (800–400)	173384	+16 %	160437	+5 %
Corr. (800–200)	67505	−55 %	260043	+70 %

**Table 3:** VPSEM-ESEM EDS analysis of NIST glass K411 (SRM 470)

Element	Certified mass fraction	Analysis 1(< 50 Pa)	Relative uncertainty (%)	Analysis 2 (650 Pa)	Relative uncertainty (%)
Mg	0.0885	0.0725	−18 %	0.058	−34 %
Si	0.2538	0.2696	+6.2 %	0.2564	1 %
Ca	0.111	0.147	+32 %	0.158	+42 %
Fe	0.112	0.148	+32 %	0.1731	+55 %
O^1^	0.424	0.363	−14 %	0.354	−17 %

**Table 4:** Composition of phases in raney nickel alloy (phases designated according to gray level in a BSE image)

	Al	Contrast Δ*C/C*	Ni	Contrast Δ*C/C*
Dark	0.988		0.0122	
Intermediate	0.588	40%	0.409	97%
Bright	0.434	26%	0.565	28%
